# Allelopathic interactions between the brown algal genus *Lobophora* (Dictyotales, Phaeophyceae) and scleractinian corals

**DOI:** 10.1038/srep18637

**Published:** 2016-01-05

**Authors:** Christophe Vieira, Olivier P. Thomas, Gérald Culioli, Grégory Genta-Jouve, Fanny Houlbreque, Julie Gaubert, Olivier De Clerck, Claude E. Payri

**Affiliations:** 1 UMR ENTROPIE, LabEx-CORAIL, U227 “Biocomplexité des écosystèmes coralliens”, Institut de Recherche pour le Développement, Nouméa, Nouvelle-Calédonie, France; 2 Phycology Research Group and Center for Molecular Phylogenetics and Evolution, Ghent University, Gent, Belgium; 3 Sorbonne Universités, UPMC Univ Paris 06, IFD, Paris, France; 4 Institut de Chimie de Nice-EEIC, UMR 7272 Université Nice Sophia Antipolis, CNRS, Faculté des Sciences, Nice, France; 5 Institut Méditerranéen de Biodiversité et d’Ecologie marine et continentale (IMBE), Aix Marseille Université, CNRS, IRD, Avignon Université, Station marine d’Endoume, rue de la Batterie des Lions, Marseille, France; 6 Université de Toulon, MAPIEM, EA 4323, La Garde, France; 7 Laboratoire de Pharmacognosie et de Chimie des Substances Naturelles- UMR CNRS 8638 COMETE - Université Paris Descartes, Paris, France

## Abstract

Allelopathy has been recently suggested as a mechanism by which macroalgae may outcompete corals in damaged reefs. Members of the brown algal genus *Lobophora* are commonly observed in close contact with scleractinian corals and have been considered responsible for negative effects of macroalgae to scleractinian corals. Recent field assays have suggested the potential role of chemical mediators in this interaction. We performed *in situ* bioassays testing the allelopathy of crude extracts and isolated compounds of several *Lobophora* species, naturally associated or not with corals, against four corals in New Caledonia. Our results showed that, regardless of their natural association with corals, organic extracts from species of the genus *Lobophora* are intrinsically capable of bleaching some coral species upon direct contact. Additionally, three new C_21_ polyunsaturated alcohols named lobophorenols A–C (**1–3**) were isolated and identified. Significant allelopathic effects against *Acropora muricata* were identified for these compounds. *In situ* observations in New Caledonia, however, indicated that while allelopathic interactions are likely to occur at the macroalgal-coral interface, *Lobophora* spp. rarely bleached their coral hosts. These findings are important toward our understanding of the importance of allelopathy versus other processes such as herbivory in the interaction between macroalgae and corals in reef ecosystems.

Like many other groups of organism macroalgae are known to influence the growth, survival, and reproduction of other organisms in their vicinity by producing allelochemicals. Early studies on macroalgal allelopathy predominantly focused on four main categories of effects: (1) regulation of algal populations, (2) regulation of invertebrate colonization, (3) lethal and sublethal effects on fishes, and (4) antimicrobial activities^1^,^2^. By far, allelopathic defensive functions against herbivores have been the most extensively studied role for macroalgal secondary metabolites over the past 30 years^3^. More recent studies also revealed the role of allelopathy in the competition with benthic competitors other than algae and notably with corals^4^. A series of studies demonstrated that some macroalgae possess allelochemicals with bleaching properties on specific coral species^5^,^6^,^7^,^8^. Allelopathy against corals has been suggested in the brown algal genus *Lobophora* J. Agardh (Dictyotales, Phaeophyceae). But while *Lobophora* exhibits a wide array of bioactivities (e.g. antibacterial, antifungal, antiviral) see^9^ for review, a limited number of studies were directed towards understanding the ecological roles of *Lobophora* natural products e.g.^8^,^10^,^11^,^12^,^13^. Nevertheless, *Lobophora* remains an important benthic component of tropical coral reefs and species of this genus are commonly observed interacting with scleractinian corals in the Caribbean^14^,^15^ and in the Pacific^16^,^17^. Among the macroalgae present in the southwestern lagoon of New Caledonia, *Lobophora* is most commonly encountered in association with scleractinian corals. A review on the species diversity in New Caledonia indicated that the genus is a lot more diverse than reported in the literature^18^ with at least 31 species, present in New Caledonia. Furthermore, species closely associated with scleractinian corals predominantly belong to a specific clade. *Lobophora* species have apparently developed very specific ecological niches together with morphologies. For instance, four species of *Lobophora* with decumbent to encrusting growth forms are in direct contact with corals (i.e. *L. hederacea*, *L. monticola*, *L. rosacea*, *L. undulata*), while other species with different morphologies were found growing in different habitats and substrates^18^. Association with corals, except in some rare cases^19^, did not represent an apparent threat for corals, but rather a shelter for algae from herbivores^20^. Nevertheless, *Lobophora* has been considered a potent competitor against corals, particularly following the dramatic regime shift in the Caribbean and Great Barrier Reef^16^,^21^,^22^,^23^. Subsequently, several studies have aimed at studying *Lobophora-*coral interactions and understanding the mechanisms by which species of *Lobophora* may outcompete corals. Dead coral surface is generally a prerequisite for the algal settlement while only a limited number of living coral species seem vulnerable to *Lobophora* overgrowth^24^,^25^,^26^,^27^. However, two studies showed that *Lobophora* allelochemicals presented bleaching properties against three coral species, *Porites astreoides*, *P. cylindrica* and *Montastraea cavernosa*^8^,^11^. Conversely, one study demonstrated that *Lobophora* waterborne compounds enabled coral recruitment^12^. Overall, *Lobophora* association with corals has been largely interpreted as negative, even though only a limited number of studies convincingly demonstrated that *Lobophora* could pose an important threat to corals.

Taking into account that: (1) some *Lobophora* species are naturally occurring associated with coral species on healthy reefs without apparent signs of competition towards their coral “hosts”, and; (2) that *Lobophora* organic extracts displayed allelopathy against some coral species in bioassay experiments, we address the following questions: Do *Lobophora* species naturally found in association with corals present negative allelopathy against the latter; are all *Lobophora* species, regardless of their association with corals, equally susceptible to bleach corals; and last, if allelopathic interactions are at play, which compounds mediate these interactions? To tackle these questions, we implemented a multi-level approach of allelopathic bioassays starting from a multi-species and crude extract level to a single species and isolated compounds level. We first tested and compared allelopathy effects of several species of *Lobophora* crude extracts against several species of corals. Then, we compared the negative allelopathy of numerous semi-purified fractions and purified compounds from a single *Lobophora* species on the most vulnerable coral.

## Results

### Importance of *Lobophora*–corals associations in New Caledonia

Association between *Lobophora* and corals occurs in a variety of habitats, ranging from coral-dominated to algal-dominated communities. We monitored 78 transects in the southwest lagoon and detected *Lobophora* species associated with corals in 54 transects (69%) (Table 1). Restricting ourselves to transects in which *Lobophora* was present, the average percentage of associations of this species ranged from 7 to 24%. Three species, *L. abscondita*, *L.crassa* and *L. nigrescens* were never associated with corals. Instead these species grew on a variety of substrates such as dead coral rubble and bedrock (Table 1).

*Lobophora* species are associated with a limited number of coral genera. Association between *Lobophora* and *Acropora* is by far the most common. Except in the case of *L. hederacea* where the alga appears to have deleterious effects on the *Seriatopora* coral^19^, living parts of other corals were not overgrown by *Lobophora* nor presented evident traces of bleaching. *Lobophora* predominantly grew at the dead basal parts of branching coral colonies. In the case of *L. rosacea*, the alga forms dense rosettes niched within the coral branches. In the case of *L. hederacea* and *L. monticola* the alga attaches itself to the coral base and adopts decumbent forms, while *L. dimorpha* adopts a procumbent form.

### Effects of *Lobophora* spp. extracts on corals

**Figure 1:**
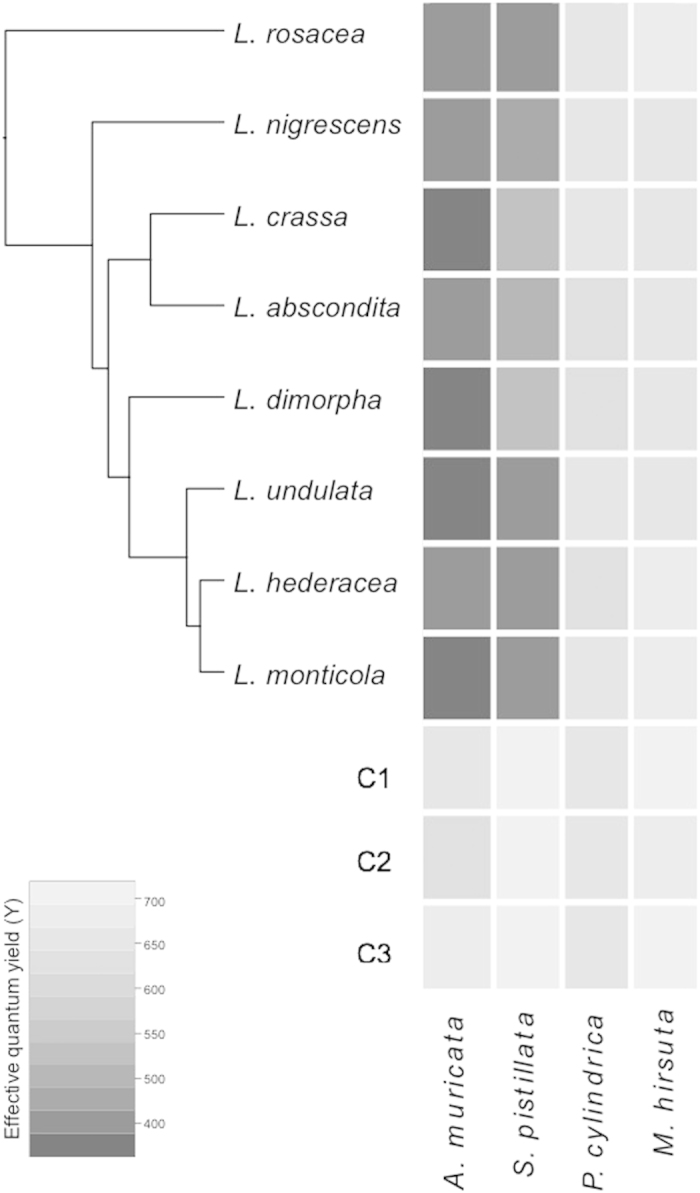
Heatmap representation of the bioassay results of eight species of *Lobophora*, viz. *L*.*rosacea*, *L. nigrescens*, *L. crassa*, *L. abscondita*, *L. dimorpha*, *L. undulata*, *L. hederacea* and *L. monticola*, crude extracts tested against four coral species, viz. *Acropora muricata*, *Stylophora pistillata*, *Porites cylindrica* and *Montipora hirsuta*. The color is indicative of the coral effective quantum yield (Y) measurement under the patch surface after 24 h of exposure. C1 (no patch), C2 (patch without solvent) and C3 (patch with solvent) are the three controls. The phylogenetic tree is the maximum clade credibility tree obtained from BEAST analysis of the concatenated alignment of four genes (*rbc*L, *cox*3, *psb*A and LSU) from Vieira *et al.*^18^.

All extracts prepared from *Lobophora* species caused significant visual bleaching on the corals *A. muricata* and *S. pistillata* and suppression of photosynthetic efficiency *in situ*, relative to controls (*p* < 0.001), while no significant bleaching effects were detected in *P. cylindrica* and *M. hirsuta* (Fig. 1). In general, *A. muricata* was more pronouncedly bleached than *S. pistillata* (Fig. 1). No significant difference was observed between the *Lobophora* species (Fig. 1). Consequently, *A. muricata* was selected as a target coral for the identification of allelopathic compounds, and the alga *L. rosacea* was chosen as it is the most common and abundant species in the southwest lagoon of New Caledonia, allowing collection of enough material for subsequent analytical identification of purified allelopathic compounds.

### Bioassay-guided fractionation

**Figure 2:**
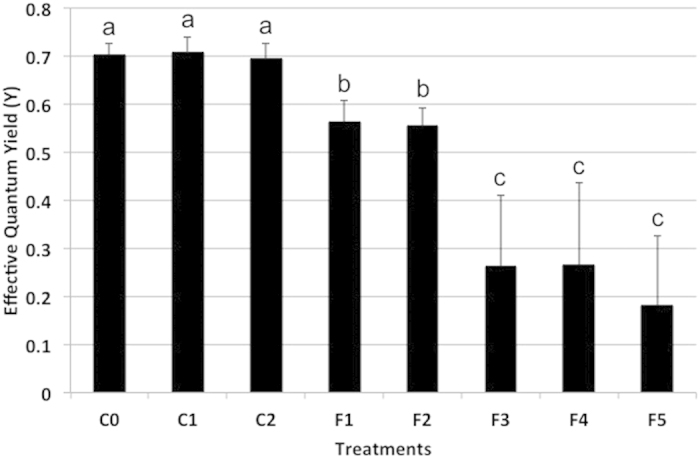
Barplot representation of the bioassays results with the five fractions of *L. rosacea* on *A. muricata*. The statistical analyses, comparing the fractions treatment patches to MeOH-treated patch and untreated patch controls, were performed using Kruskal-Wallis and Tukey’s HSD post-hoc test. Letters indicate distinct groupings based on post-hoc statistical comparison among sub-fractions. Asterisks indicate significance in relation to controls (MeOH-treated or untreated, accordingly) with P < 0.001, n = 10 assays, ≥5 fractions per assay for all experiments. Error bars represent standard deviation of the mean. C1 (no patch), C2 (patch without solvent) and C3 (patch with solvent) are the three controls.

The *L. rosacea* extract was fractionated by VLC into five fractions of contrasting polarity. Out of the five fractions tested against the coral *A. muricata*, the less polar ones (F3 to F5) caused significant visual bleaching and suppression of photosynthetic efficiency relative to controls (Fig. 2), with a decrease of the photosynthetic efficiency of ca. 50% for F3 and F4, and of 70% for F5. The most polar fractions (F1 and F2) significantly suppressed coral photosynthetic efficiency (25% decrease) but less than F3–F5. F4 and F5 displayed very similar HPLC-DAD-ELSD-MS profiles and consequently only F3 and F4 were chemically studied.

**Figure 3:**
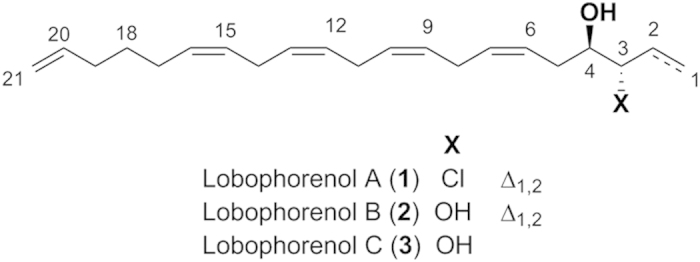
Chemical structure of Compounds 1–3. Compound **1** (F3P13): (6Z,9Z,12Z,15Z)-nonadeca-1,6,9,12,15,18-hexaene-3,4-diol; Compound **2** (F3P10a): (6Z,9Z,12Z,15Z)-nonadeca-6,9,12,15,18-pentaene-3,4-diol; Compound **3** (F3P11b): (6Z,9Z,12Z,15Z)-4-chlorononadeca-6,9,12,15,18-pentaen-3-ol.

A first fractionation of F3 by reversed phase HPLC resulted in 14 sub-fractions named F3P1 to F3P14. Because most of them were still identified as mixtures of compounds by ^1^H NMR, the most bioactive sub-fractions were further purified to identify compounds responsible for the bioactivity. Therefore, the final purification of F3P13, F3P10 and F3P11 led to the pure compounds **1** (F3P13a), **2** (F3P10a) and **3** (F3P11b) respectively (Fig. 3). The structure of the chemical components of the other sub-fractions was not identified due to the low amount available or complexity of the mixture. Reversed phase HPLC fractionation of F4 resulted in five sub-fractions (F4P1-F4P5) from which no pure compound was identified.

**Figure 4:**
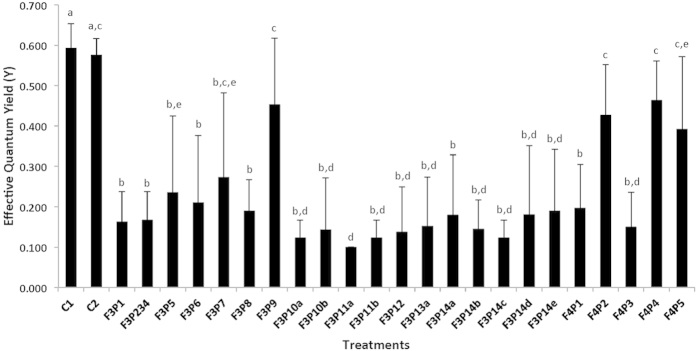
Barplot representation of the allelopathic bioassay results with the 23 compounds isolated from the fractions 3 and 4 of *L. rosacea* on *A. muricata*. The statistical analyses, comparing the compounds-treated patchs to MeOH-treated patch and untreated controls, were performed using Kruskal-Wallis and Steel-Dwass-Critchlow-Fligner post-hoc test. Letters indicate distinct groupings based on post-hoc statistical comparison among sub-fractions. Asterisks indicate significance in relation to controls (MeOH-treated or untreated, accordingly) with P < 0.001, n = 10 assays, 23 sub-fractions per assay. Error bars represent standard deviation of the mean. Letters indicate significant differences (Kruskal–Wallis test, P < 0.01; Steel–Dwass post-hoc test, P < 0.05, mean + s.d., n = 10).

Subfractions and pure compounds caused contrasting effects, with ca. 80% of them causing significant bleaching and suppression of photosynthetic efficiency relative to controls (Fig. 4). The suppression of photosynthetic efficiency ranged from ca. 40 to 80%, relative to the coral effective quantum yield baseline, depending on the sub-fractions. Based on the Tukey HSD post hoc test results, six significantly different groups of allelopathic sub-fractions or pure compounds stood out. Three allelopathic compounds were selected for structure identification, as they were considered sufficiently pure.

### Structure identification of compounds 1–3

**Figure 5:**
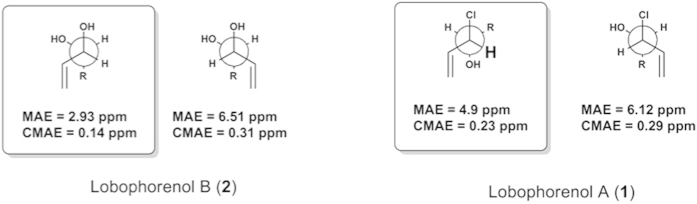
Mean Absolute Errors (MAE) and Corrected Mean Absolute Errors (CMAE) obtained between the ^13^C NMR experimental and theoretical values for the two possible diasteroisomers of compounds 1 and 2.

Compound **1** was isolated as colorless oil and its molecular formula was proposed as C_21_H_31_ClO by HRESIMS analysis ([M + NH_4_]^+^ at *m/z* 352.2407 and 354.2382 with isotopic ratio 3:1). The ^1^H NMR analysis started with a terminal vinyl group at *δ*_H_ 5.34 (dt, H-1a), 5.21 (dt, H-1b) and 6.02 (ddd, H-2) which was COSY coupled to a deshielded methine at *δ*_H_ 4.38 (ddt, H-3) (Table 2). Even if we first suspected the presence of a secondary alcohol at this position, the chemical shift of the corresponding carbon was more shielded than expected at *δ*_C_ 67.8 (C-3) for an allylic alcohol. In agreement with MS data, we then deduced the presence of a chlorine atom at this position which was COSY correlated to a oxygenated methine (*δ*_H_ 3.70 ddd, H-4; *δ*_C_ 75.2, C-4). The spin coupled system was then extended to an ABXM system at *δ*_H_ 2.49 (H-5a) and 2.25 (H-5b) which was further coupled to an alternate polyunsaturated carbon chain composed of four double bonds separated by three methylenes. The configurations of the double bonds were assigned as *Z* by interpretation of the chemical shifts of allylic carbons. All these connections were later confirmed using HSQC and HMBC spectra. The other end of the compound was deduced to be composed of a second terminal vinylic system coupled to the polyunsaturated core trough three COSY correlated methylene units. Unfortunately no similar allylic chlorohydrine was found in the literature that could allow us to conclude on the relative configuration of **1**. We then decided to compare the ^13^C NMR experimental values with the calculated values obtained on the most stable conformers of the *like* and *unlike* diasteroisomers. Working on the most stable conformer, the Mean Absolute Error (MAE) was found to be lower for the *unlike* configuration (Fig. 5).

For **2**, the isotopic pattern of the HRESIMS spectrum evidenced the absence of a chlorine atom in this molecule and the molecular peak at *m/z* 334.2744 ([M + NH_4_]^+^) suggests the replacement of this atom by an alcohol. Inspection of the ^1^H and ^13^C NMR spectra allowed us to localize the structural changes in the vicinity of the first vinylic system. Indeed, the methine signals at *δ*_H_ 4.38 (ddt, H-3) and *δ*_C_ 67.8 (C-3) in **1** were replaced by signals at *δ*_H_ 3.94 (H-3) and *δ*_C_ 76.6 (C-3) that are reminiscent of an allylic secondary alcohol. Therefore, the chlorine atom placed at C-3 in **1** was replaced by a second alcohol in **2** at this position. The relative configuration of compound **2** was deduced to be *unlike* applying the same method as for **1** (Fig. 5). In this case, hydrogen bonds between the two vicinal alcohols render the *gauche* conformer more stable than the *anti* obtained for **1**. Both compounds may be produced by an *anti* opening of a common epoxide intermediate with water or a chloride ion.

The HR-(+)ESIMS data obtained for compound **3** with a molecular peak at *m/z* 336.2895 ([M + NH_4_]^+^) suggested that this natural product corresponds to a dihydrogenated derivative of **2**. The location of the reduced double bond was unambiguously deduced from ^1^H NMR data that showed the lack of a terminal vinylic system. The appearance of a methyl at *δ*_H_ 0.97 (t, H-1) definitely placed the new ethyl group at the beginning of the chain. We assume the same relative configuration for this compound as those previously proposed for **1** and **2**, being linked biosynthetically. The low amounts of compounds isolated prevented any attempts to assign their absolute configuration at C-3 and C-4.

## Discussion

**Figure 6:**
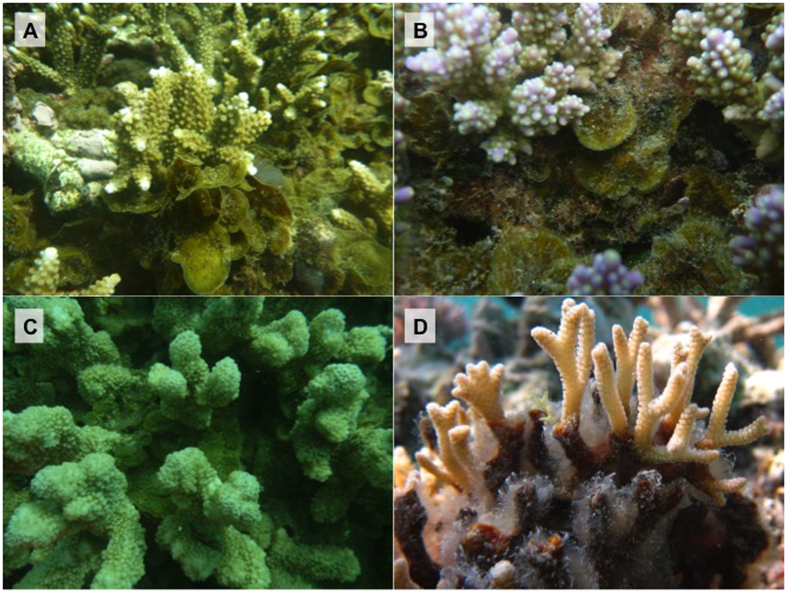
Pictures of natural association between *Lobophora* spp. and coral species in New Caledonia. (**A**) *L. rosacea* at next to *Acropora* sp., (**B**) *L. undulata* at the base of *Acropora* sp., (**C**) *L. rosacea* at the bases of *Acropora lobata*, (**D**) *L. hederacea* on *Seriatopora caliendrum* branches. Photo credit: Christophe Vieira.

Proliferation of *Lobophora* in coral reef environments has repeatedly caused concern among biologists^16^,^28^. There is, however, considerable uncertainty regarding the causes underlying such proliferations as well as of the threat that *Lobophora* poses to corals. In addition, given the recent progress in understanding species-level diversity in the genus, it is not known whether all or only a subset of *Lobophora* species compete with corals for space. Our survey of *Lobophora* – coral associations in the southwest lagoon of New Caledonia demonstrates that not all species of *Lobophora* associate with corals. Out of eight *Lobophora* species, three never associated with a coral, but instead grew attached to scattered hard substrate in seagrass beds, shallow wave-washed habitats or coral rubble. Nevertheless, perhaps some interactions may have been detected with greater observations. The other five species were associated with living coral colonies, but grew at the dead bases adopting procumbent to decumbent forms (e.g. *L. dimorpha, L. undulata, L. hederacea, L. monticola*), or a fasciculate morphology niched within coral branches such as *L. rosacea*. The latter species is also the most commonly encountered, being observed in 42% of the transects. Most *Lobophora* species, however, were only observed in 10 to 20% of the transects. Even then, these numbers tend to overestimate the prevalence of *Lobophora* on the entire reef since the sites where the transects were laid out were precisely those locations where *Lobophora* – coral interactions were most conspicuous during initial surveys. Per transect, the presence of *Lobophora* never surmounted 25%. *Acropora* species were clearly the preferred partner, but all but one *Lobophora* species displayed a broader range of hosts. Corals associated with *Lobophora* did not present traces of bleaching (Fig. 6), except in the case of *L. hederacea* associated with *Seriatopora caliendrum*.

Based on the ecological niche and the morphological differentiation between *Lobophora* species we investigated if the species found in direct contact with corals have developed specific allelochemicals capable of impairing corals. Our results demonstrate that all *Lobophora* species, usually found in contact or not with corals, displayed similar bleaching effects on the tested corals. In other words, naturally found in contact or not, extracts of the eight *Lobophora* species show similar effects on corals: they are equally capable or not of bleaching specific corals. These results are of significant importance as it implies that species of the genus *Lobophora* are intrinsically capable of bleaching some corals upon direct contact. In an evolutionary context, this either means that *Lobophora* has developed: (1) allelopathic compounds targeted towards competing benthic organisms or (2) allelopathy against corals, or other benthic organisms, may be a side-effect (i.e. a secondary unintentional effect) of secondary metabolites with different ecological roles, such as antimicrobial properties (e.g. biofilm deterrents). Recent findings by Rasher and Hay^29^ showing that the red alga *Galaxaura filamentosa* uses different compounds to compete with corals versus to resist herbivores would refute the side-effect hypothesis. However, it is unknown at present if a differentiation between allelochemicals and anti-herbivory chemicals is the rule rather than the exception. At least the large fraction of pure or mixed compounds (80% of the isolated compounds from two fractions) that result in a significant suppression of photosynthetic efficiency on corals, would argue against such differentiation.

Among the four coral species tested*, A. muricata* and *M. hirsuta* were the most significantly bleached corals. These results indicated a differential susceptibility to *Lobophora* allelopathy depending on the coral species. In this aspect our results echoed those of Rasher *et al.*^5^ who also noticed differential susceptibility across coral species to algal allelopathy. Rasher *et al*.^5^ found that *A. millepora* and *P. damicornis* were more sensitive to macroalgal allelopathic damage than *M. digitata* and *P. cylindrica*. We shared three genera (*Acropora, Montipora*, and *Porites*) and one species (*P. cylindrica*) with Rasher *et al.*^5^. Also, we had similar results across those genera, although in our case *M. hirsuta* and *P. cylindrica* were not damaged at all. *P. damicornis*, which belongs to the same family as *S. pistillata*, i.e., Pocilloporidae, was also quite sensitive^5^. However, *Acropora* and *Montipora*, which belong to the same family (Acroporidae), were differentially susceptible in both studies. Partly agreeing with our findings, Lesser *et al.*^30^ showed that Acroporids are not as resilient in the face of environmental perturbation compared to other species on the same reef. Nugues and Bak^27^ also showed that Caribbean corals had differential competitive abilities against *Lobophora*.

We then proceeded to the isolation and structure identification of the chemicals from *L. rosacea* exhibiting bleaching properties against *A. muricata*, the most susceptible coral out of the four tested. Results from the bioassays with the five fractions showed that allelopathy against coral correlates with the polarity of the compounds, with the less polar fractions displaying the highest allelopathic activity. These results concur with the findings of Rasher and Hay^8^, showing that lipidic extracts from several algal species, including *Lobophora variegata*, resulted in significant bleaching, while hydrophilic compounds from *Chlorodesmis fastigata* (Udoteaceae, Chlorophyta) and *Galaxaura filamentosa* (Galaxauraceae, Rhodophyta) were not active. These results corroborate the importance of direct contact, which is preferable for hydrophobic allelochemicals transfer.

Most of the purified compounds from *L. rosacea* displayed a significant bleaching effect on *A. muricata*. The three new C_21_ polyunsaturated alcohols, named lobophorenols A–C (**1**–**3**) were among the most active fractions and sub-fractions were identified after NMR and MS analyses. These compounds were identified as three new C_21_ polyunsaturated alcohols. All these compounds may originate after opening of a common epoxide intermediate formed from a polyene. Similar C_21_ apolar polyenes have been reported only once from the alga *Fucus vesiculosus*^31^. It is worth highlighting the presence of a chlorinated analogue **1**, which is particularly rare and represent less than 1% of all the secondary metabolites isolated from species of the Phaeophyceae family^32^,^33^. Although, we may point out that De Nys *et al.*^8^ also isolated halogenated allelochemicals, the presence of the chlorine atom may however not be related to the bleaching properties of the molecule, since both compounds **2** and **3**, deprived of this halogen atom, present similar adverse properties. Furthermore, the isolated allelochemicals do not belong to the terpene family of natural products, as somewhat expected from De Nys *et al.*^6^ and Rasher and Hay^5^ but polyunsaturated alcohols. It shows that allelopathy against corals may involve a variety of families of compounds as already reported by Slattery and Lesser^11^, and strongly supported by the diversity of compounds displaying bleaching properties in this study. The lack of ability to correlate bioactivity with classes of compounds is well known and been discussed numerous times for anti-herbivore compounds, antibiotics, etc.^34^,^35^,^36^. It is worth pointing out that we were expecting to find terpenes, given the richness in terpenoids of the Dictyotaceae family to which *Lobophora* belongs^37^. However, much to our surprise this family of compounds was not detected by NMR. Yet, the genus *Zonaria*, which is sister to *Lobophora*, did not present terpenes either (authors’ unpublished data).

*Lobophora* bioactivity against corals does not come as a surprise as in the literature *Lobophora* extracts (crude, hydrophilic or hydrophobic extracts) and isolated compounds have been shown to display a broad spectrum of activities and in particular antimicrobial (e.g. fungi, bacteria, protozoa) bioactivities e.g.^10^,^38^,^39^. The exact bleaching mechanisms are unaddressed here and could very well be targeting either the polyp or the *Symbiodinium*. Nonetheless, it is worth mentioning that after two weeks following the bioassays, the surface area which bleached in contact with the patches, recovered their original coloration.

Present field assays would suggest that *Lobophora* has the potential to chemically impair some coral species by direct contact. Nevertheless, *in situ* observations indicate that although apparently chemically potent, *Lobophora* do not or rarely bleach coral hosts in a natural setting (this study). Slattery and Lesser^11^ also questioned if *Lobophora* presented allelopathic effects on corals in the Bahamas. Yet, while *Lobophora* extracts and a purified compound bleached the coral *Montastrea cavernosa*, contact experiments between *Lobophora* and the coral did not^11^. Furthermore, no claim of coral bleaching as a result of contact with *Lobophora* in natural setting was made by the authors^11^. Even though it would be tempting to conclude that allelopathy is ecologically important in the competition between *Lobophora* and corals, there is no strong evidence from field observations. Herbivory on the other hand, clearly appears as an important factor preventing competition to occur^11^,^17^,^26^,^28^. Therefore, the question remains: what explains the inconsistency between field observations and bioassay experiments? A possible explanation for this discrepancy would be the localization of the bioactive compounds within the endometabolome. Bioassays artificially expose corals to chemicals, a situation that would only occur as a result of abrasion or herbivory under natural conditions. Alternatively, the compounds may be part of the exometabolome, present on the surface of the alga, but external factors (e.g. herbivory) or a defense system by the coral itself may ward off allelopathic interactions, thereby preventing *Lobophora* from outcompeting corals. Several investigators have demonstrated that bioactive lipids distributed on the surfaces of algae, including *Lobophora*, were capable of damaging corals^5^,^7^,^8^,^40^. This forms a strong indication that at least some compounds are present on the algal surface. Given the presence of allopathic compounds, most coral species prevent the overgrowth of crustose *Lobophora* species owing to a set of defense mechanisms^24^,^27^. Additionally, field observations and experiments showed that herbivory is a major factor preventing increase in *Lobophora* abundance^11^,^26^,^28^. In New Caledonia, only one species of *Lobophora*, *L. hederacea*, was observed overgrowing a coral species, *Seriatopora caliendrum*^19^. In the latter case, coral overgrowth appears to be possible owing to a combination of factors including the coral vulnerability and the inhibition of grazing^19^, supporting the important role of coral defense and herbivory in preventing negative allelopathic interactions. In the Great Barrier Reef, Jompa and McCook^17^ showed that a crustose *Lobophora* species was capable of overgrowing the coral *Porites cylindrica* when herbivory was reduced. In damaged reefs, however, coral morbidity and mortality in addition to shifts in herbivory pressure result in whole different setting where macroalgal allelopathy may have harmful effects on corals. Although not yet explored, it is possible that allelopathy in damaged reef may results from the synergetic effects of macroalgal exudates/allelochemicals acting in combination with a number of environmental parameters/stressors such as seawater pH, oxygen depletion, and or temperature maxima^1^.

The role of chemical interactions between macroalgae and corals initially evinced in the early 90s in form of positive allelopathy^41^, has regained interest only recently, yet this time in form of negative allelopathy^8^. Most studies on the subject have disclosed deleterious effects (e.g. bleaching, recruitment inhibition) in damaged reefs. The present work, focusing on a healthy reef ecosystem, provided evidence that allelopathic defense is not restricted to *Lobophora* species that are naturally found in close contact with corals. These findings are important toward our understanding of the importance of allelopathic competition and defense systems versus herbivory in the interaction between macroalgae and corals in reef ecosystems.

## Materials and methods

### Quantification of *Lobophora*–corals association

Eight species of *Lobophora*, commonly encountered in the southwest lagoon of New Caledonia were selected to quantify their association with corals and for the bioassays, i.e. *L. abscondita, L. crassa, L. dimorpha, L. hederacea, L. monticola, L. nigrescens*, *L. undulata*, and *L. rosacea* (Fig. 6). 78 belt transects, as described by English *et al.*^42^, each 10 m long, were deployed across coral dominated reefs in the southwest lagoon of New Caledonia. Within a belt transect, a 2500 cm^2^ quadrat (50 × 50 cm) was placed consecutively left and right along a defined line, and photographs were taken directly above each quadrat using a Lumix Panasonic digital camera (12 megapixels) mounted on a photoquadrat framer. In each quadrat, the frequency of *Lobophora* – coral associations was assessed by placing 16 points per quadrat using a stratified random point count method using the software “Coral Point Count with Excel extensions”^43^. Details of the sampling locations and quantification methods are outlined in Supplementary information. From these data we calculated the percentage of transects in which *Lobophora* was associated with corals. The average percentage of associations of each species was calculated per transect where the species was observed.

### Preparation of the extracts and fractions of *Lobophora* for bioassays

Algal samples for bioassays were collected by SCUBA in January 2013 in the southwest lagoon of New Caledonia (Supplementary information). Samples were cleaned from epiphytes and stored at −20 °C until freeze-drying. Four coral species were selected as targets of the bioassays, i.e. *Acropora muricata* (Linnaeus, 1758; Acroporidae), *Montipora hirsuta* (Nemenzo, 1967; Acroporidae), *Stylophora pistillata* (Esper, 1797; Pocilloporidae) and *Porites cylindrica* (Dana, 1846; Poritidae). Algal specimens were identified at species-level using mitochondrial *cox*3 gene sequences see^18^. The area of each individual was estimated using the aluminum foil technique^44^. Then, the specimens were freeze-dried and the dried samples were ground with a mortar and pestle using liquid nitrogen. One gram of ground powder was exhaustively extracted, by adding consecutively three times 10 mL of a 1:1 mixture of dichloromethane/methanol (CH_2_Cl_2_/MeOH) (v/v), leaving it 5 min in an ultrasonic bath and 5 min to decant, and then retrieving the supernatant liquid (upper phase) using a 100 mm in diameter and 10 μm in porosity qualitative filter paper folded (Whatman, UK). The resulting supernatant was concentrated under vacuum and the extracts were weighted and divided by the algal surface area to obtain a mass of extract per surface area (μg.cm^−2^).

Crude extracts of *L. rosacea* were then submitted to fractionation in order to gain information on the polarity of the compounds responsible for the allelopathic activity. The dried extract was resuspended in MeOH/CH_2_Cl_2_ (1:1; v/v), mixed with an equal amount of C_18_ silica powder (Polygoprep^®^ 60-50, Macherey-Nagel, France) and concentrated under vacuum. The resulting powder was deposited on a solid phase extraction (SPE) cartridge (Strata^®^ C18-E, 500 mg/6 mL; Phenomenex, USA) and then fractionated using five solvent mixtures (10 mL for each) of decreasing polarity: H_2_O, H_2_O/MeOH (1:1; v/v), MeOH, MeOH/CH_2_Cl_2_ (3:1; v/v), and then MeOH/CH_2_Cl_2_ (1:1; v/v). The five resulting fractions (F1 to F5) were evaporated under a fume hood during 48h, weighted, and divided by the algal surface area to obtain a quantity of fraction per surface area (μg.cm^−2^).

### Isolation and structure identification of specialized metabolites

Since no *Lobophora* species stood out in terms of bioactivity against *A. muricata* or any of the other corals (cf. results), *L. rosacea* was chosen for subsequent analytical identification of purified allelopathic compounds as it is the most common and abundant species in the southwest lagoon of New Caledonia, thus allowing collection of enough material for subsequent analytical identification of purified allelopathic compounds. The biomass (209 g of dry mass) of *L. rosacea* was exhaustively extracted, by adding consecutively five times MeOH/CH_2_Cl_2_ (1:1, v/v; 1.2 L of solvent), leaving it 10 min in an ultrasonic bath and 5 min to decant, and then retrieving the supernatant liquid. The resulting extract was concentrated under vacuum to yield a homogeneous dry powder (8.3 g). The extract was then mixed with an equal amount of C_18_ silica powder (Polygoprep^®^ 60-50) and fractionated by Vacuum Liquid Chromatography (VLC) into five fractions (F1–F5), eluting with the five organic solvents aforementioned for SPE. An additional elution was done with CH_2_Cl_2_ in order to ensure exhaustive compounds extraction from the crude extract, and was additionally tested as a sixth fraction (F6). The resulting filtrates were evaporated under vacuum, resuspended into MeOH to reach a concentration of 10 mg∙mL^−1^, filtered through 0.22 μm PTFE syringe filters (Phenomenex, UK) and filled into HPLC vials for subsequent Ultra-High Performance Liquid Chromatography-Diode Array Detection (UHPLC-DAD) analyses and High Performance Liquid Chromatography (HPLC) purification.

According to the results on the ecological activity (cf. next paragraph), F3 and F4 were selected for compounds isolation and purification. The HPLC purification was performed on a Jasco (Groß-Umstadt, Germany) preparative HPLC system (pump PU-2087 plus; diode array detector MD 2018 plus; column thermostat CO 2060 plus; autosampler AS 2055 plus; LC Net II ADC Chromatography Data Solutions; sample injection loop: 250 μL) on a phenyl-hexyl reversed phase column (XSelect CSH™, 5 μm, 19 × 250 mm; Waters, France), using for F3 an isocratic elution mode [acetonitrile (CH_3_CN) + 0.1% trifluoroacetic acid (TFA)/H_2_O + 0.1% TFA; 69/31, v/v] and a flow rate of 10 mL/min. Fourteen sub-fractions (from F3P1 to F3P14) were obtained. Fraction F4 was fractionated on the same column with a CH_3_CN/H_2_O + 0.1% TFA gradient on a 30 min run (0–5 min: 90% CH_3_CN; 5–10 min: 90 at 100% CH_3_CN, 10–25 min: 100% CH_3_CN) at 10 mL/min, leading to five sub-fractions (F4P1 to F4P5). The purification of compounds from four sub-fractions of F3 (F3P10, F3P11, F3P13, and F3P14) were performed on a C_18_ semi-preparative column (XSelect CSH™ C_18_, 5 μm, OBD, 19 × 250 mm; Waters, France) with a CH_3_CN/H_2_O + 0,1% TFA gradient (UV detection: 210 nm, flow rate: 10 mL/min).

Among all the fractions and sub-fractions only the three major, pure and bioactive compounds **1**–**3**, corresponding to fractions F3P13a (18.4 mg), F3P10a (3.8 mg) and F3P11b (3 mg) respectively, were identified on the basis of NMR and MS data.

NMR analyses were performed in CD_3_OD on a Bruker Avance 500 spectrometer using signals of the residual peaks of the solvent for calibration of the chemical shifts in ppm (*δ*_H_ 3.31 for ^1^H NMR and *δ*_C_ 49.0 for ^13^C NMR). LC-DAD-ELSD-ESI/MS^n^ analyses were carried out on a LaChrom Elite HPLC (VWR-Hitachi) composed of a L-2130 quaternary pump, a L-2200 autosampler, and a L-2300 column oven. Detection was performed with a L2455 DAD and an ELSD (Chromachem model, Eurosep) coupled to an Esquire 6000 spectrometer. UHPLC-HRMS were performed on a UHPLC U3000 (Dionex) coupled to a QqToF Impact II (Bruker).

### *In situ* allelochemical assays

Field experiments, conducted *in situ* were designed to keep the coral under natural field conditions, thus limiting pre-experimental stress usually resulting from cutting, gluing and transplantation. The bioassays were conducted in Sainte Marie Bay (22° 17.863′ S, 166° 28.898′ E) with three of the coral genera, i.e. *Acropora muricata, Porites cylindrica* and *Montipora hirsuta* and on genus in Maitre Islet Reef for *Stylophora pistillata* (22° 20.446′ S, 166° 24.108′ E). A series of three bioassay experiments were successively performed. The first experiment evaluated the bioactivity of the crude extract of the *Lobophora* species previously selected (*L. abscondita*, *L. crassa*, *L. dimorpha*, *L. hederacea*, *L. monticola*, *L. nigrescens*, *L. undulata*, and *L. rosacea*) on four coral species (*Acropora muricata*, *Porites cylindrica*, *Stylophora pistillata* and *Montipora hirsuta*). The second experiment tested the bioactivity of the five fractions obtained from the extracts of *L. rosacea* on *A. muricata*. The final experiment tested the bioactivity of the sub-fractions and compounds from two of the most bioactive fractions of *L. rosacea* identified in the previous experiment (F3 and F4). All bioassay experiments were performed *in situ* directly on coral colonies at approximately natural concentration (i.e. concentration per surface area previously estimated), the latter being critical for bioassays assessing allelopathic interactions. Thereto, we determined the amount of crude extracts, fractions, sub-fractions, and pure compounds per unit of algal surface area (i.e. 1 cm^2^) and reported it to the surface of the agarose patch applied on the coral (i.e. 2 cm^2^).

A replicate was defined by one colony of coral on which all the extracts, fractions or sub-fractions (including in some cases pure isolated compounds) were tested. A total of 10 replicates were implemented. The methodology was adapted from Rasher and Hay^8^. The chemical samples (crude extracts, fractions, sub-fractions or pure compounds) were resuspended in 1 mL MeOH and added at natural concentration into a 4% agarose gel (Conda Pronadisa, Spain). The mix chemical sample/agarose was poured into a polyvinyl chloride mold, composed of 10 times 2-cm^2^ wells. Before that, tulle bands, of 20 × 2 cm, were disposed at the bottom of the wells onto which the gel mixture will adhere while gelifying. The strips were prepared the day before field application and refrigerated until then at 5 °C. They were applied onto the coral by knotting the tulle bands to the branches, and removed after 24 h of exposure. Agarose strips with and without MeOH were additionally made as controls, to ensure the non-effect of either the agarose strips itself or the solvent on the coral. Gel strips were applied on the corals between 09:00 and 11:00 AM.

### Coral photosynthesis measurements

Pulse Amplitude Modulated (PAM) fluorometry measurements were performed with a Diving-PAM (Walz) right after removal of the strips. PAM fluorometry measures the photosynthetic efficiency of photosystem II within the endosymbiotic *Symbiodinium* spp. that may be used as a quantitative measure of photo-inactivation during coral bleaching^45^. PAM fluorometry values of healthy corals are ranging between 0.5 to 0.8, depending on the coral species and time of the day. Values between 0 to 0.2 are indicative of severe bleaching or mortality^46^. As outlined in Rasher and Hay^8^ PAM fluorometry measurements were performed where the strips were applied and 5-cm next to it, as a spatial control to have a coral health baseline for comparison.

### Statistical analyses

Normality of distribution of the coral responses for all the bioassay experiments was tested with the normality Shapiro-Wilk test. If the responses violated parametric assumptions, coral responses were evaluated using the Kruskal-Wallis H test followed by the Tukey honestly significant difference (HSD) post hoc comparisons test for significant Kruskall-Wallis findings. If the data respected the parametric assumptions, a one-way ANOVA was performed followed by the Tukey post hoc HSD test for significant ANOVA findings. Statistical analyses were performed using the computing environment R^47^.

## Additional Information

**How to cite this article**: Vieira, C. *et al.* Allelopathic interactions between the brown algal genus *Lobophora* (Dictyotales, Phaeophyceae) and scleractinian corals. *Sci. Rep.* **6**, 18637; doi: 10.1038/srep18637 (2016).

## Supplementary Material

Supplementary Information

## Figures and Tables

**Table 1:** Association of *Lobophora* species with corals in the southwest lagoon of New Caledonia.

	Percentage of transects^a^	Average associations^b^	*Acropora*	*Montipora*	*Stylophora*	*Porites*	*Seriatopora*	*Turbinaria*	Non-coral substrate
*L. abscondita*	9	0	0	0	0	0	0	0	100
*L. crassa*	9	0	0	0	0	0	0	0	100
*L. dimorpha*	11	15	100	0	0	0	0	0	0
*L. hederacea*	23	23	15	0	6	22	42^c^	15	0
*L. monticola*	11	24	82	12	0	6	0	0	0
*L. nigrescens*	9	0	0	0	0	0	0	0	100
*L. rosacea*	42	22	45	22	15	12	0	0	0
*L. undulata*	19	7	50	42	8	0	0	0	0

^a^percentage of transects where the species were observed in the vicinity of corals.

^b^average percentage of associations as assessed by the stratified random point count method in transects where the species was present.

^c^*Lobophora* - coral associations with visible deleterious effects (bleaching and or overgrowth).

**Table 2:** 1H (500 MHz) and ^13^C NMR (125 MHz) chemical shifts (in ppm) for compounds 1–3 in CD_3_OD.

Compound	Lobophorenol A (1)		Lobophorenol B (2)		Lobophorenol C (3)	
	*δ*_C_	*δ*_H_, mult. (*J* in Hz)	*δ*_C_	*δ*_H_, mult. (*J* in Hz)	*δ*_C_	*δ*_H_, mult. (*J* in Hz)
1a	118.4	5.34, dt (17.0, 1.0)	116.7	5.31, dt (17.0, 1.0)	10.8	0.97, t (7.5)
1b		5.21, dt (10.0, 1.0)		5.18, dt (10.0, 1.0)		
2	137.3	6.02, ddd (17.0, 10.0, 8.5)	139.4	5.92, m	26.8	1.57, m
						1.47, m
3	67.8	4.38, ddt (8.5, 4.5, 1.0)	76.6	3.94, m	76.2	3.32, m
4	75.2	3.70, ddd (9.5, 5.5, 4.5)	75.5	3.48, m	74.9	3.45, m
5a	32.8	2.49, br dt (14.5, 5.5)	31.8	2.36, m	32.2	2.36, dt (14.5, 7.0)
5b		2.25, ddd (14.5, 8.0, 5.5)		2.14, m		2.24, dt (14.5, 7.0)
6	126.4	5.48, m	127.3	5.51, m	127.6	5.52, m
7	131.4	5.49, m	130.8	5.45, m	130.6	5.45, m
8	26.8	2.87, t (6.0)	26.7	2.85, m	26.8	2.87, t (6.0)
9	128.7	5.37, m	128.7	5.37, m	128.7	5.37, m
10	129.4	5.37, m	129.4	5.37, m	129.4	5.37, m
11	26.6	2.86, t (6.0)	26.6	2.86, m	26.6	2.86, t (6.0)
12	129	5.37, m	129	5.37, m	129	5.37, m
13	129.1	5.37, m	129.1	5.37, m	129.1	5.37, m
14	26.6	2.82, t (6.0)	26.6	2.82, m	26.6	2.82, t (6.0)
15	129.3	5.37, m	129.3	5.37, m	129.3	5.37, m
16	130.8	5.38, m	130.8	5.38, m	130.8	5.38, m
17	27.6	2.10, br q (7.0)	27.6	2.07, m	27.6	2.08, m
18	30.1	1.46, quint (7.0)	30.1	1.46, quint (7.0)	30.1	1.46, quint (7.0)
19	34.4	2.07, br q (7.0)	34.4	2.06, br q (7.0)	34.4	2.07, m
20	139.8	5.82, ddt (17.0, 10.0, 7.0)	139.8	5.82, ddt (17.0, 10.0, 7.0)	139.8	5.82, ddt (17.0, 10.0, 7.0)
21a	115.1	5.00, dq (17.0, 2.0)	115.1	5.00, dq (17.0, 2.0)	115.1	5.00, dq (17.0, 2.0)
21b		4.94, dt (10.0, 1.0)		4.94, dt (10.0, 1.0)		4.94, dt (10.0, 1.0)
