# Supplementary material for: Allelopathic interactions between the brown algal genus Lobophora (Dictyotales, Phaeophyceae) and scleractinian corals
Source: Sci Rep. 2016 Jan 5;6:18637. doi: 10.1038/srep18637 (PMC4700470; doi:10.1038/srep18637)
Supplement: Supplementary Information [file srep18637-s1.pdf]

Supplementary Information

Allelopathic interactions between the brown algal genus *Lobophora* (Dictyotales, Phaeophyceae) and scleractinian corals

Christophe Vieira, Olivier P. Thomas, Gérald Culioli, Grégory Genta-Jouve, Fanny Houlbreque, Julie Gaubert, Olivier De Clerck, Claude E. Payri

NMR spectra of compounds 1-3

HR-(+)ESIMS spectra of compounds 1-3

Assessment of the relative seafloor cover and quantification of Lobophora-corals association

Sampling locations of Lobophora species in New Caledonia

Picture of *in situ* bioassays.

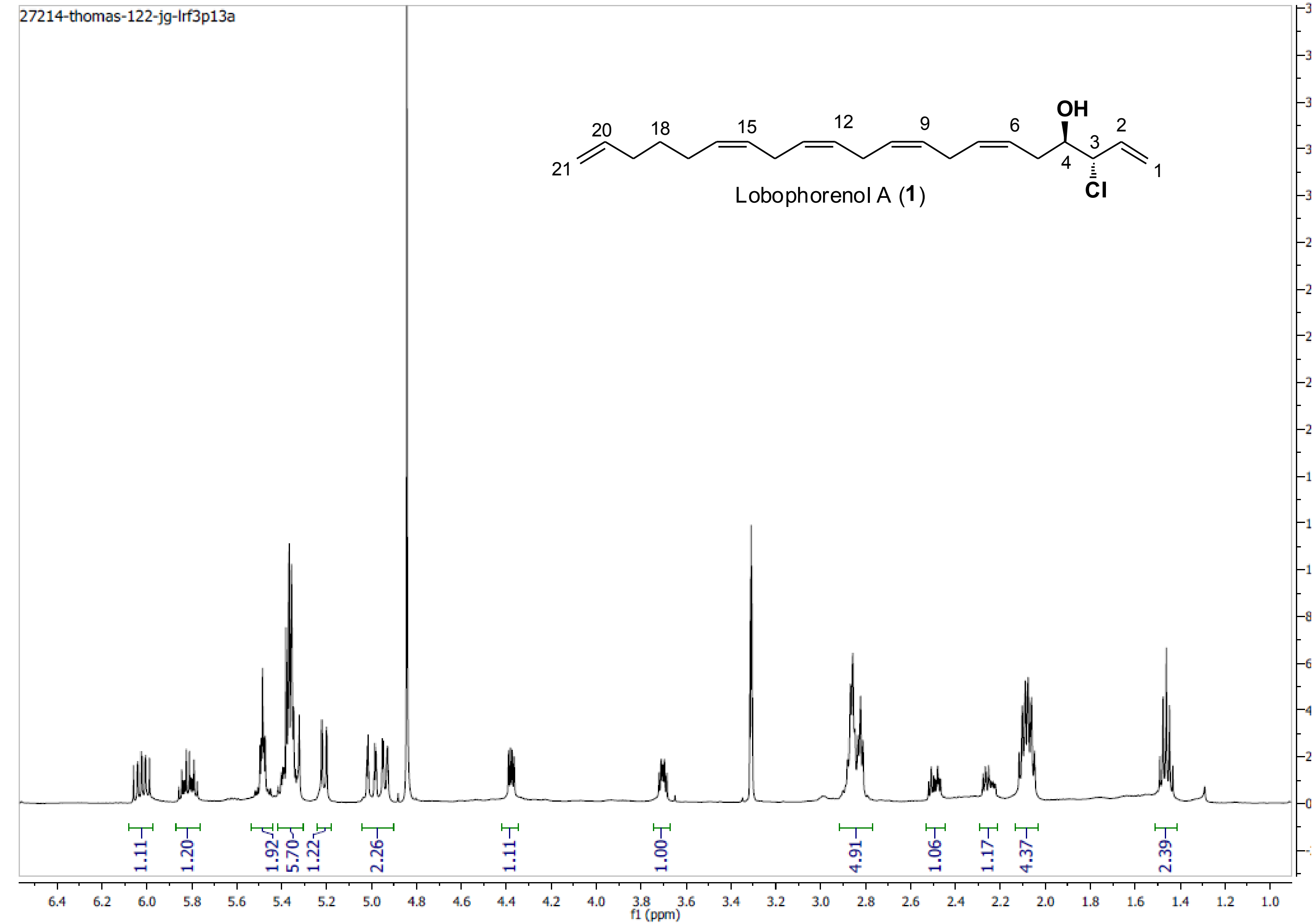

^{1}H NMR spectrum of **1** in CD_3OD at 500 MHz

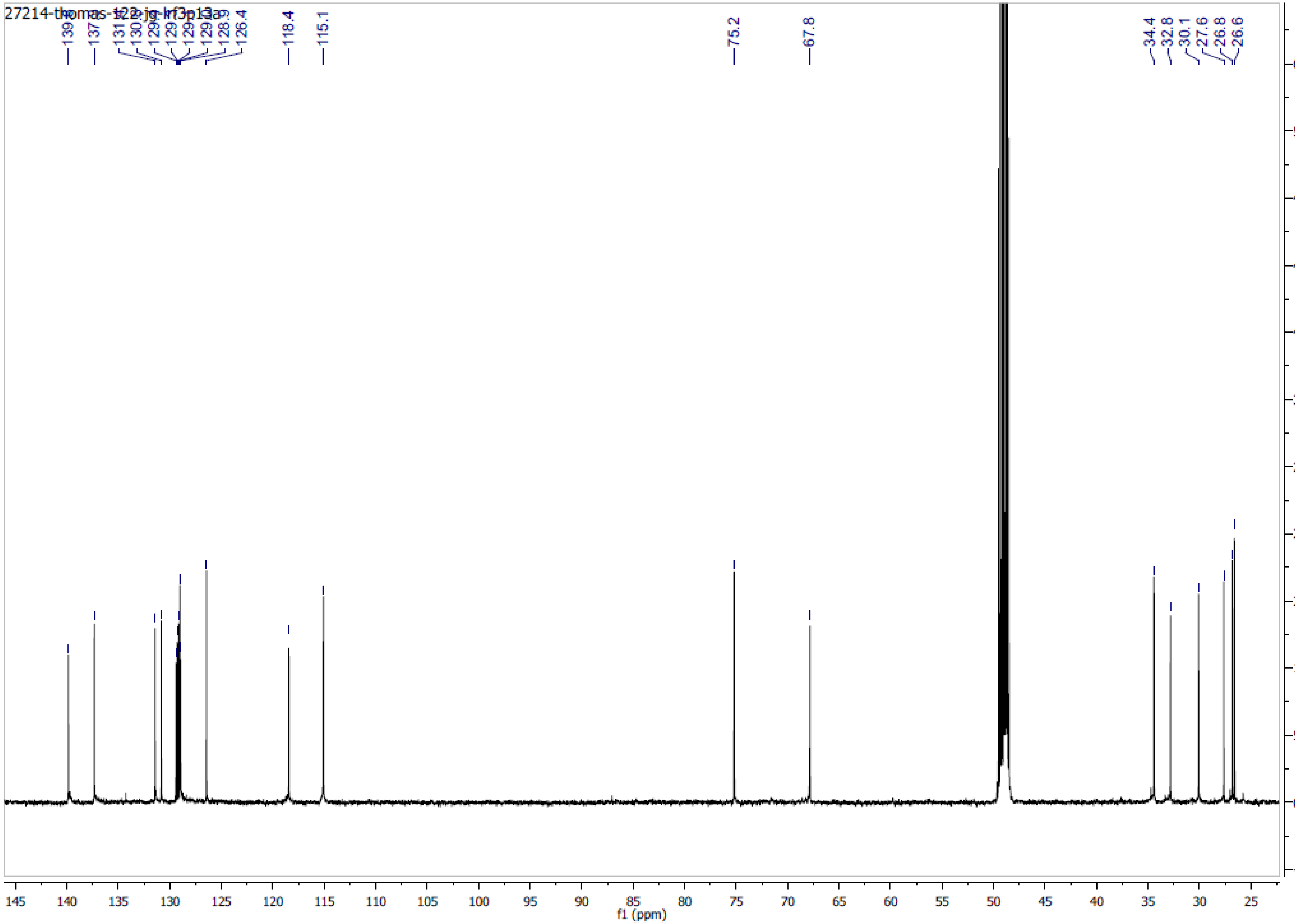

^{13}C NMR spectrum of **1** in CD_3OD at 125 MHz

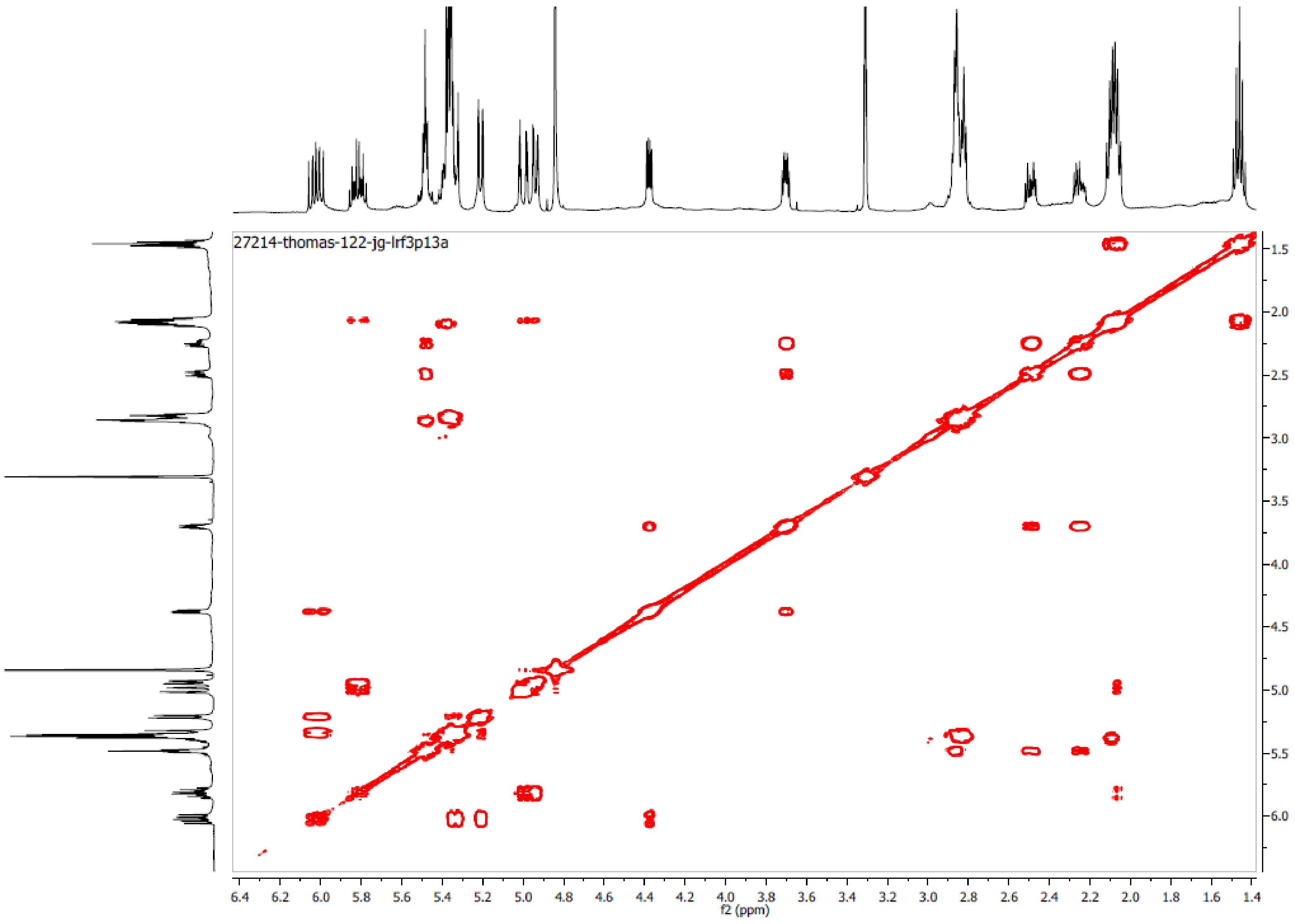

COSY NMR spectrum of **1** in CD_3OD (500 MHz)

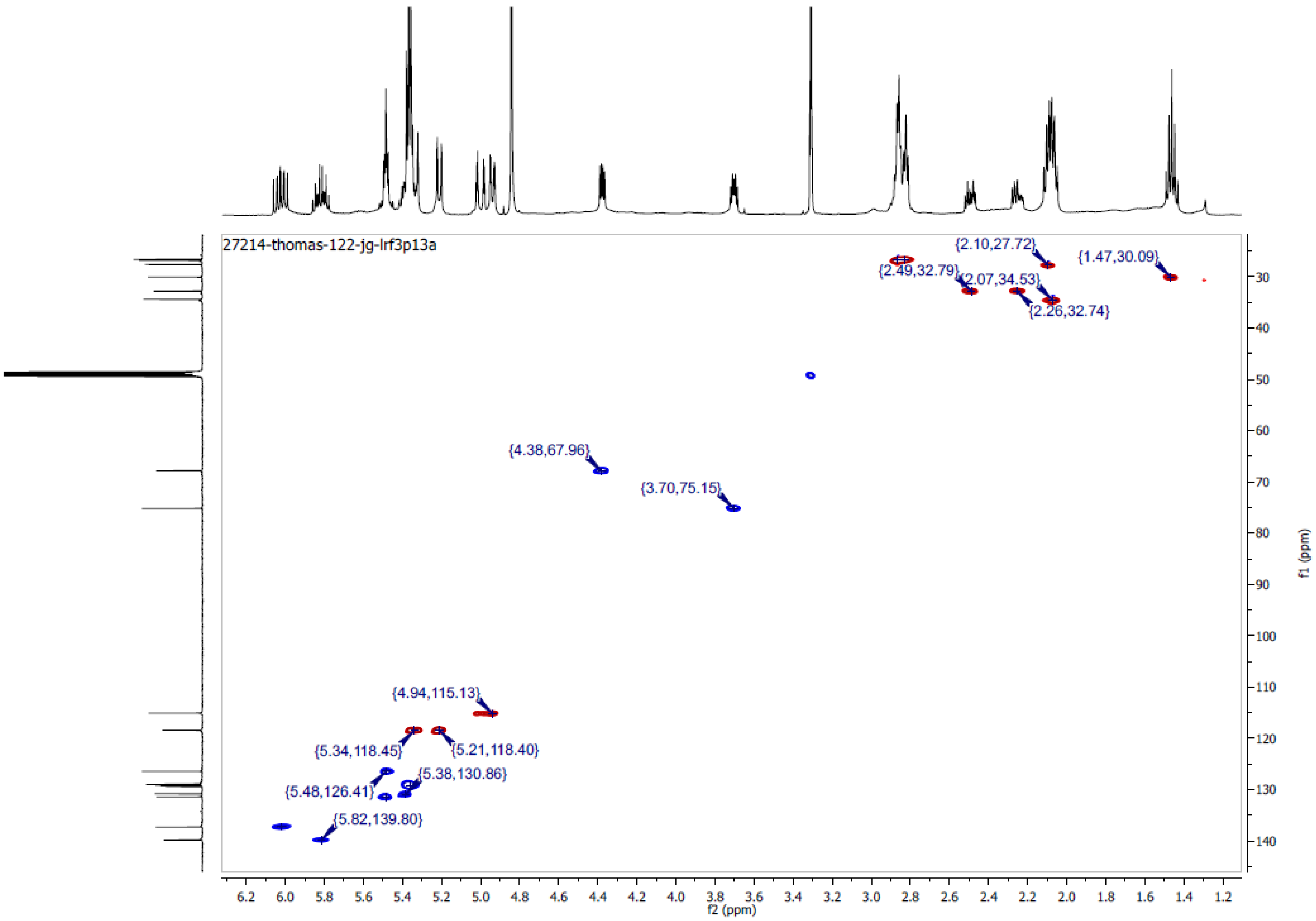

HSQC NMR spectrum of **1** in CD_3OD (500 MHz)

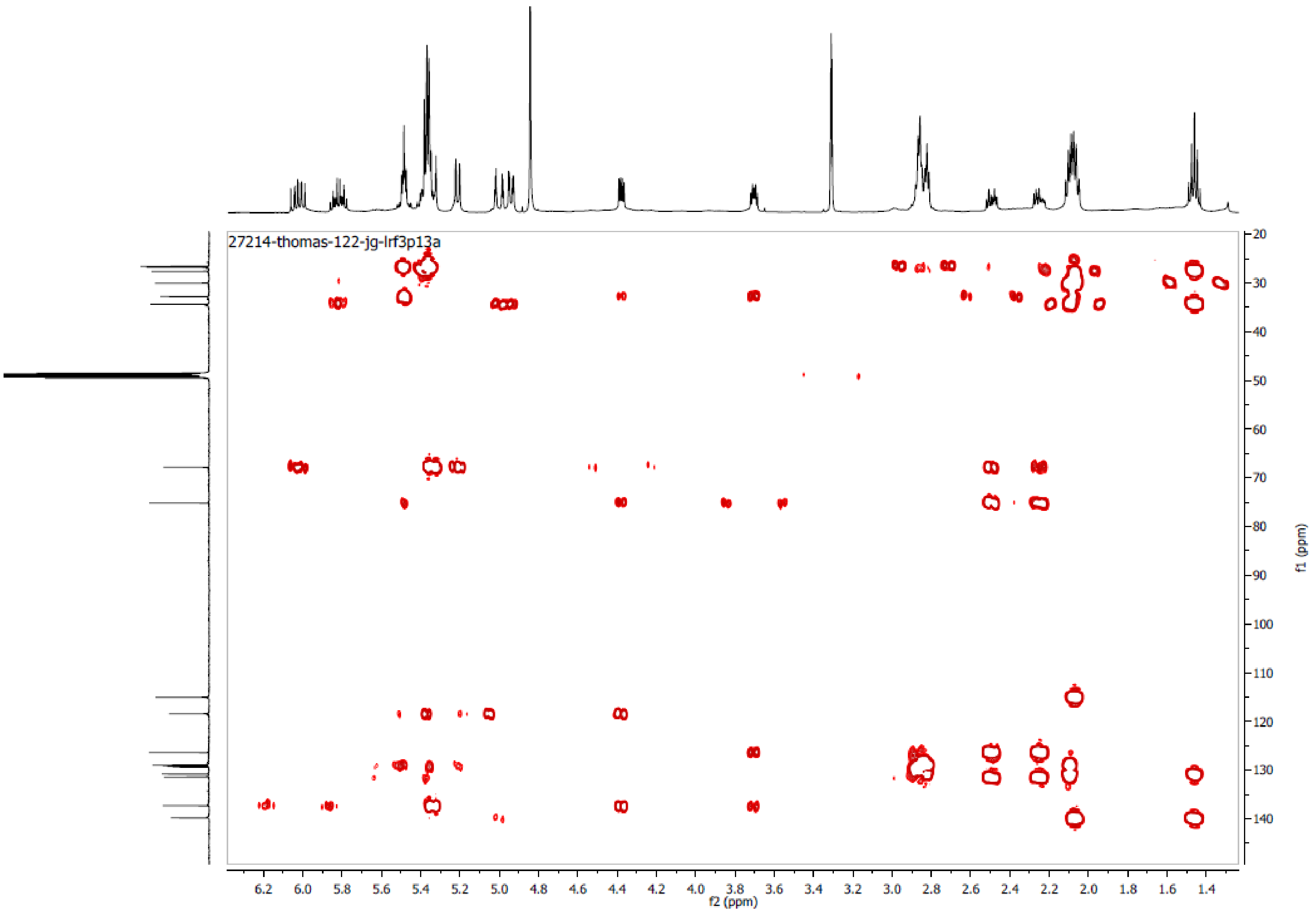

HMBC NMR spectrum of **1** in CD_3OD (500 MHz)

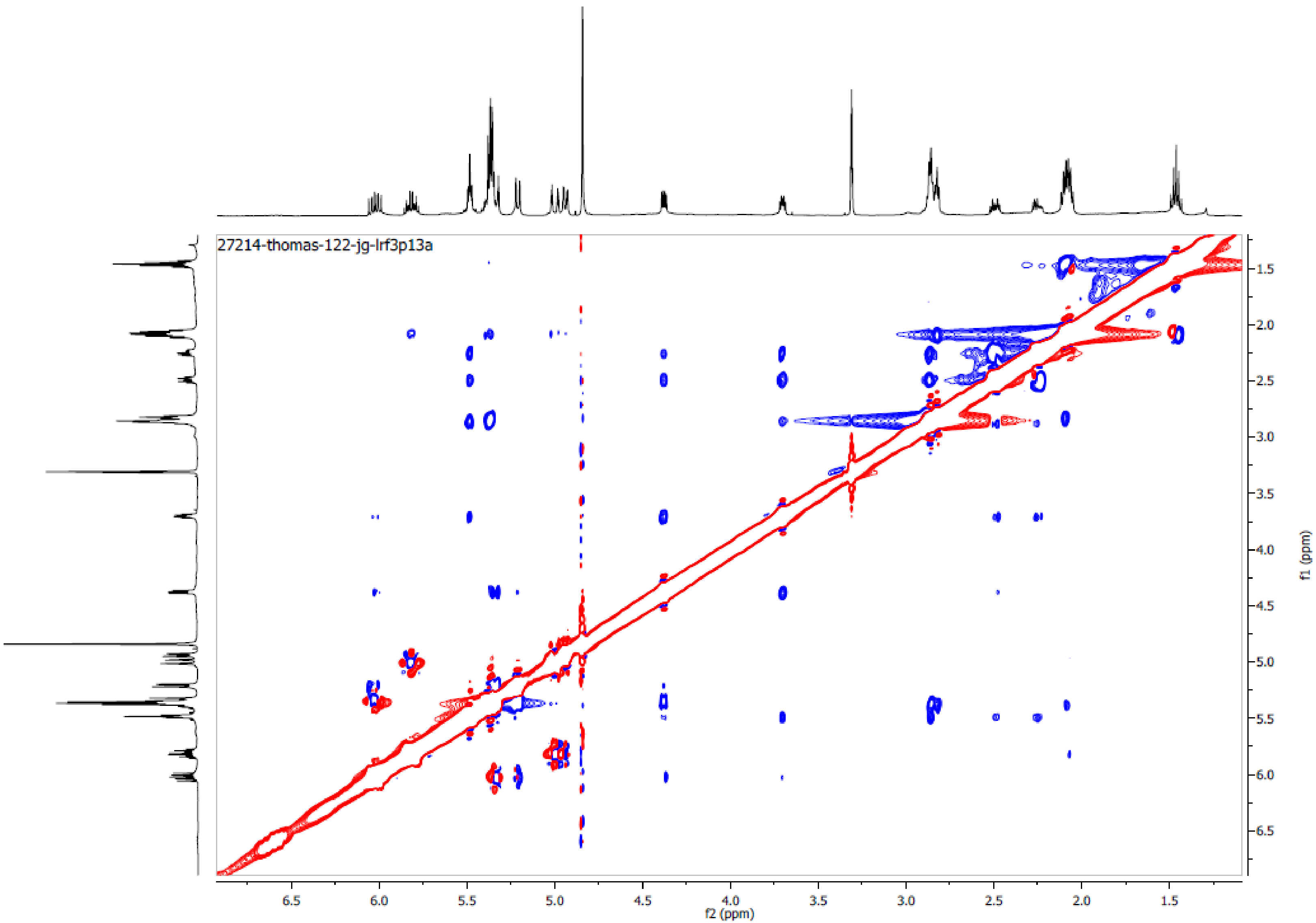

NOESY NMR spectrum of **1** in CD_3OD (500 MHz)

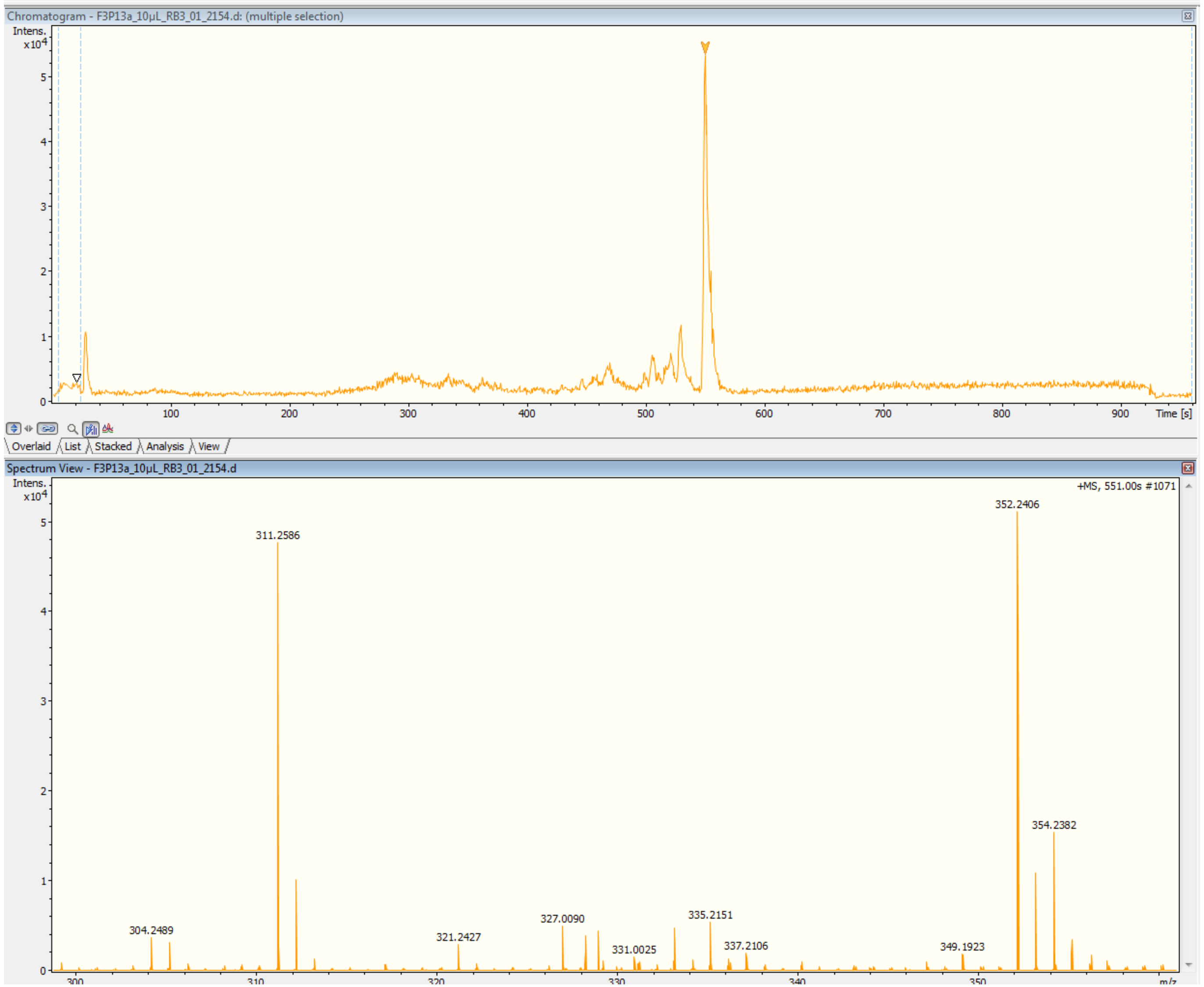

HR-(+)ESIMS spectrum of **1**

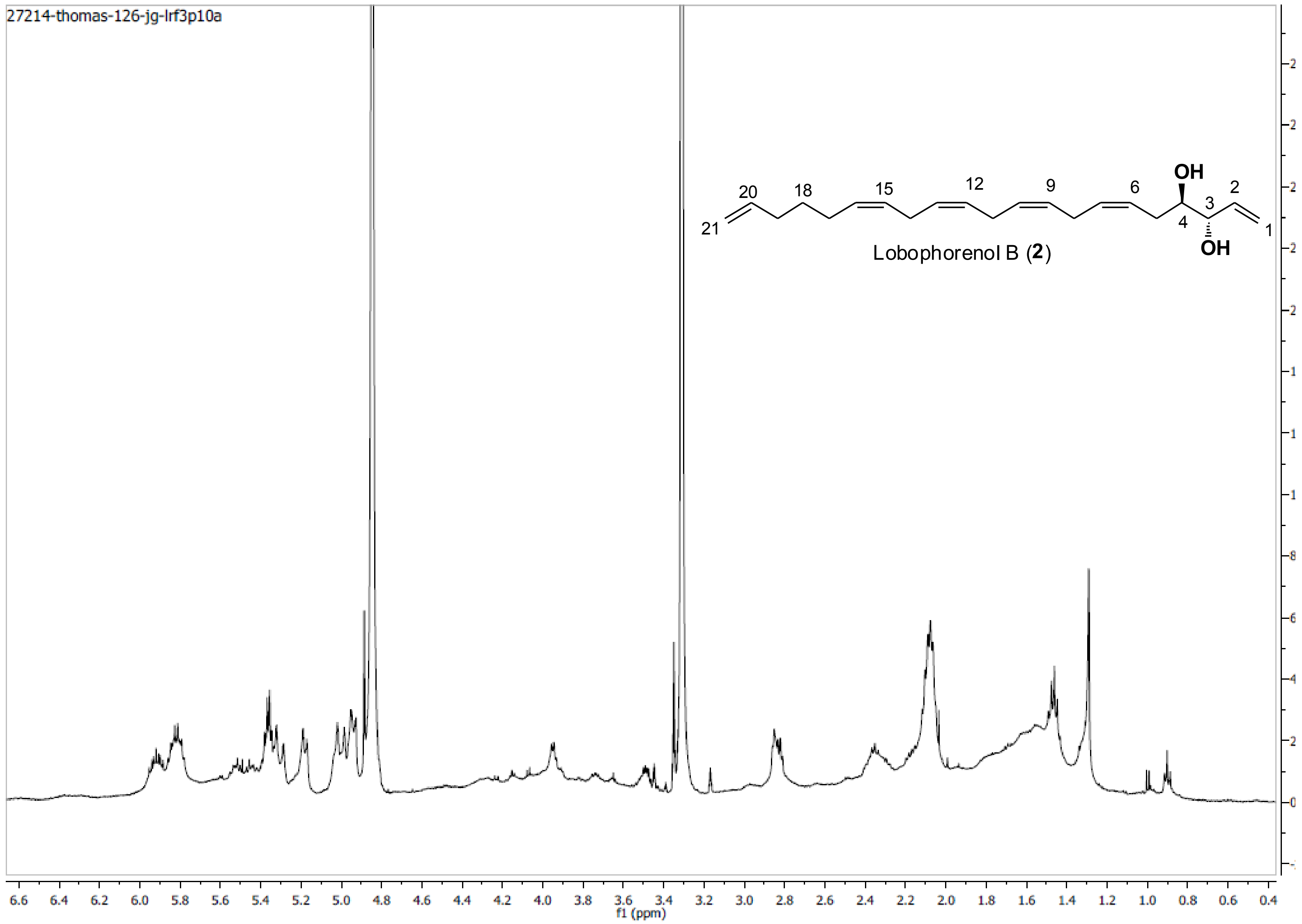

^{1}H NMR spectrum of **2** in CD_3OD (500 MHz)

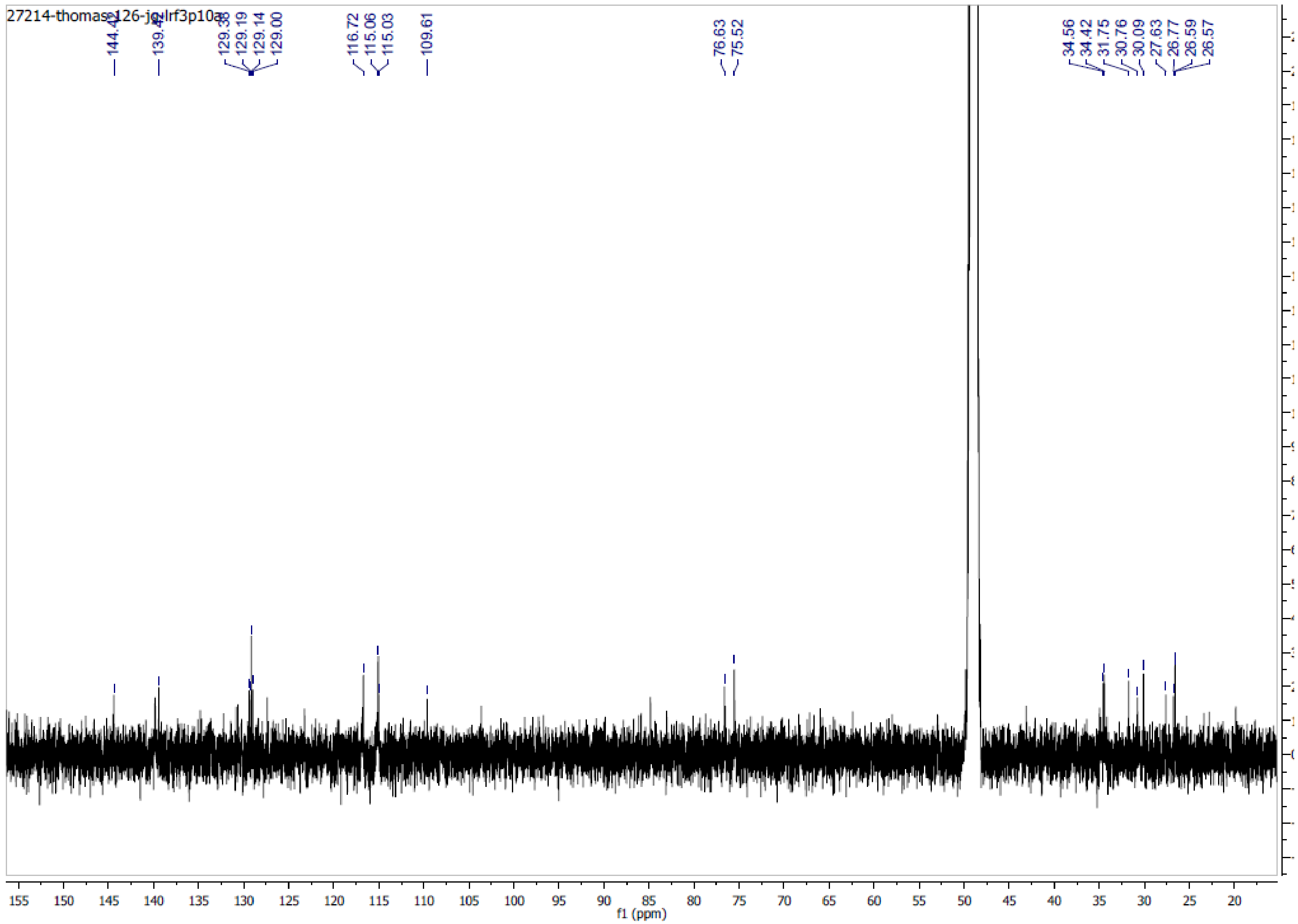

^{13}C NMR spectrum of **2** in CD_3OD (125 MHz)

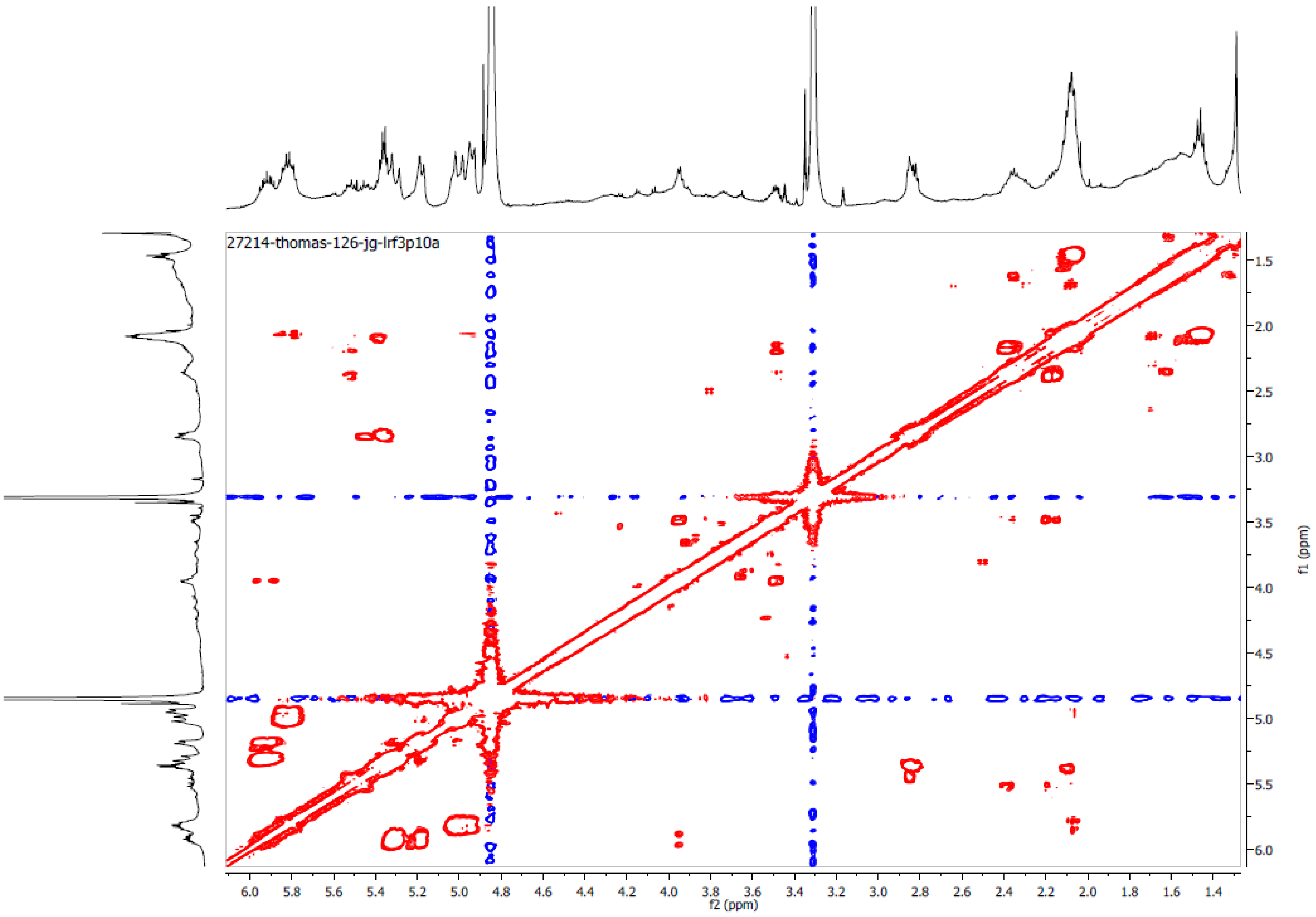

COSY NMR spectrum of **2** in CD_3OD (500 MHz)

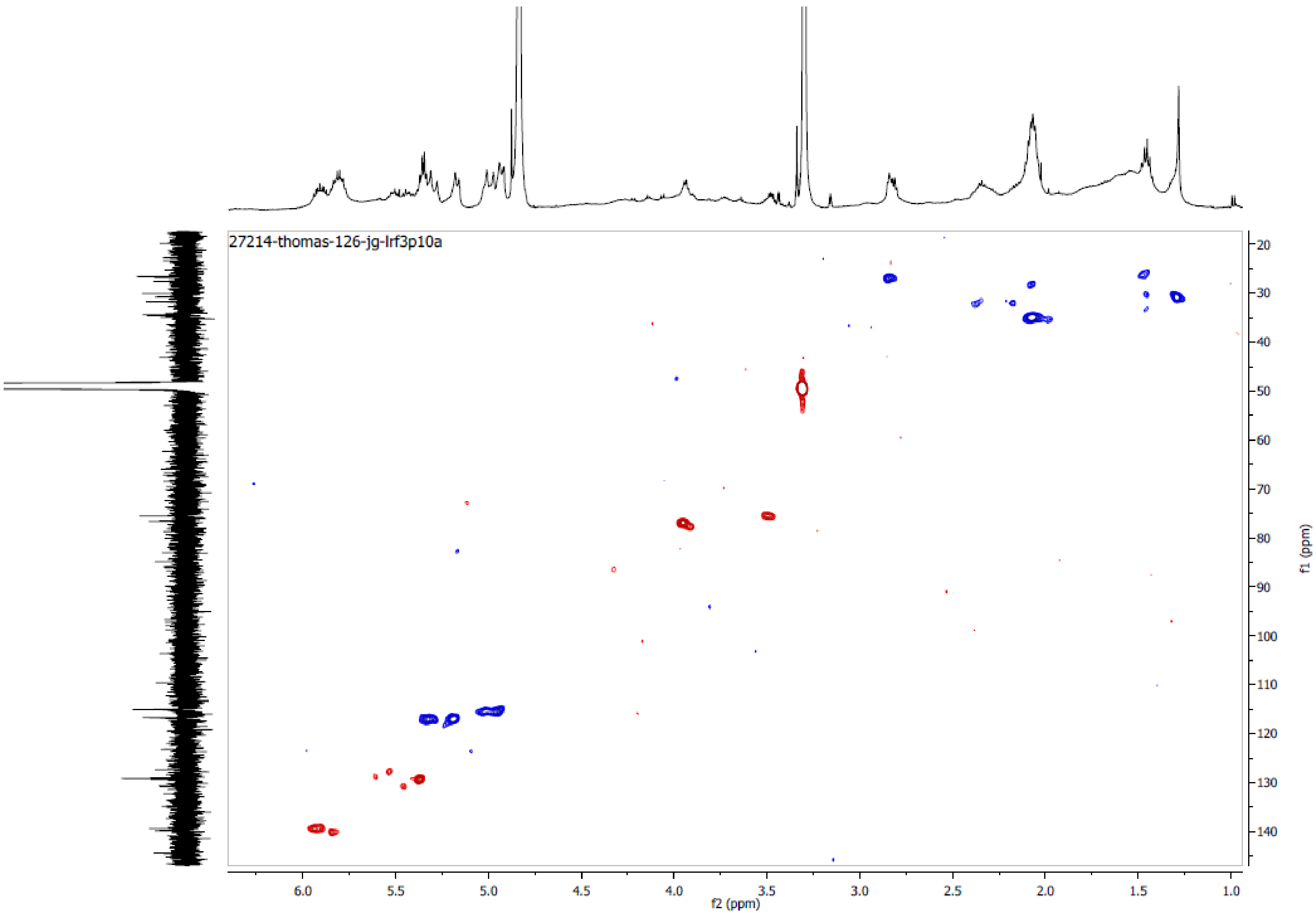

HSQC NMR spectrum of **2** in CD_3OD (500 MHz)

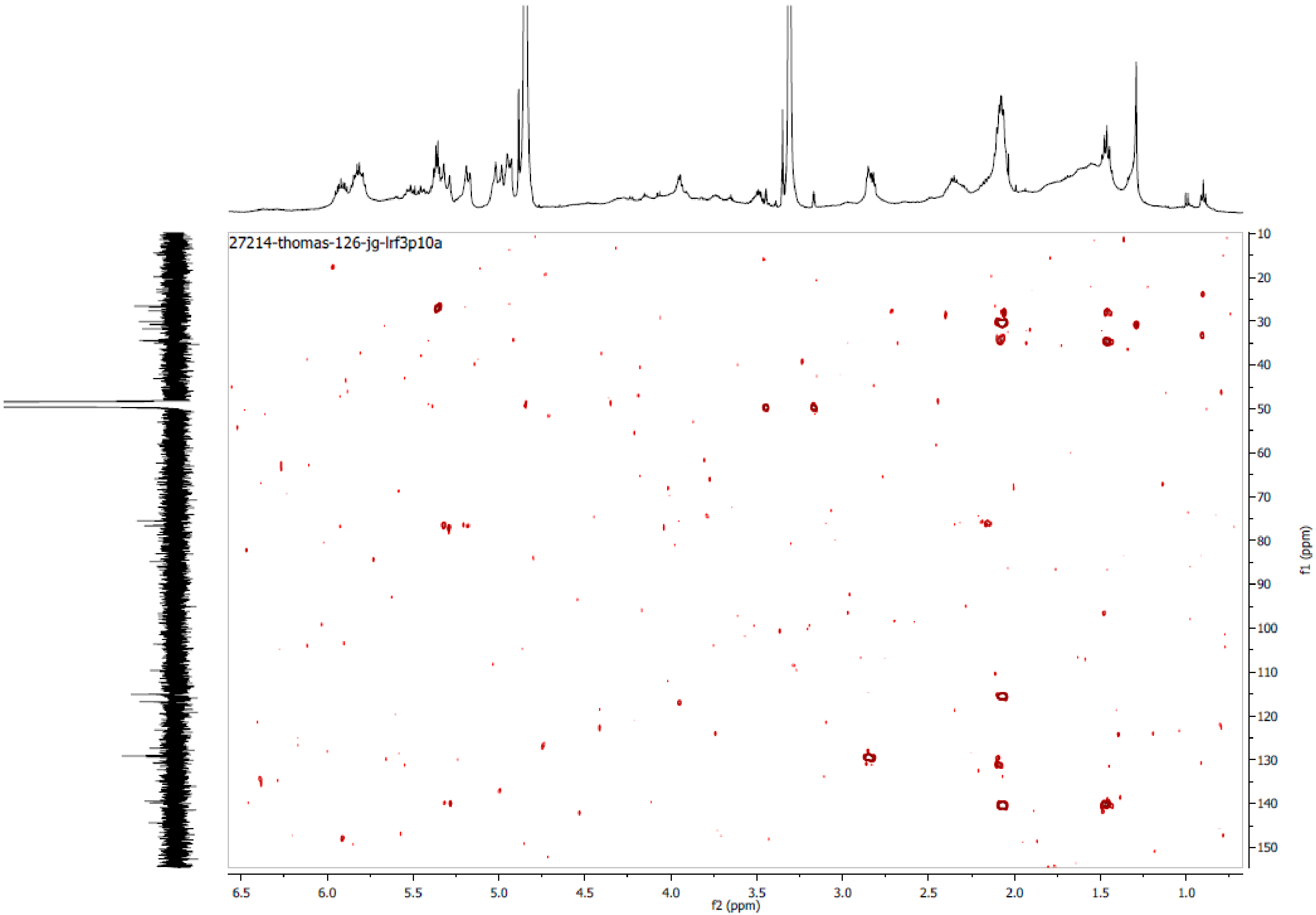

HMBC NMR spectrum of **2** in CD_3OD (500 MHz)

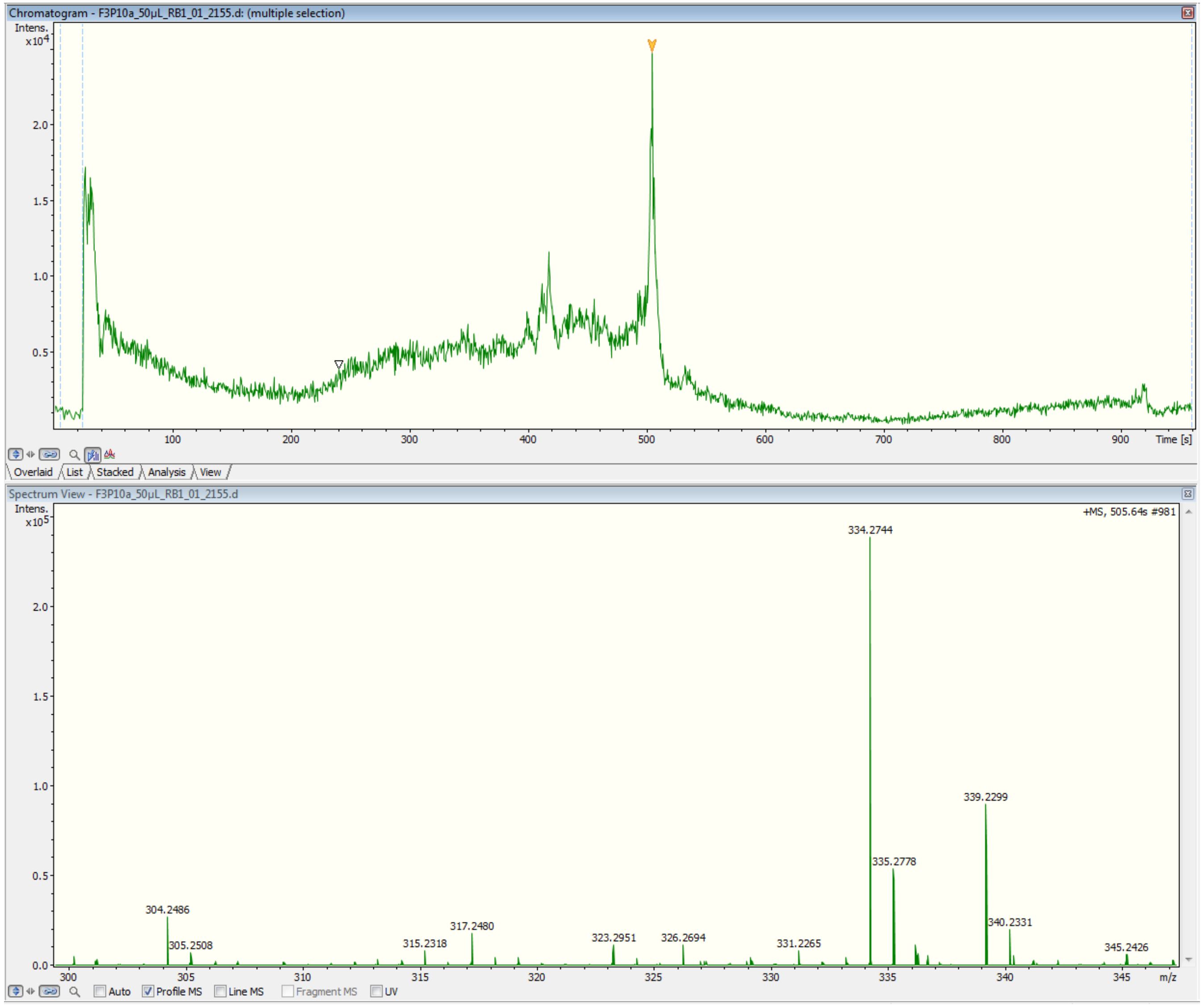

HR-(+)ESIMS spectrum of **2**

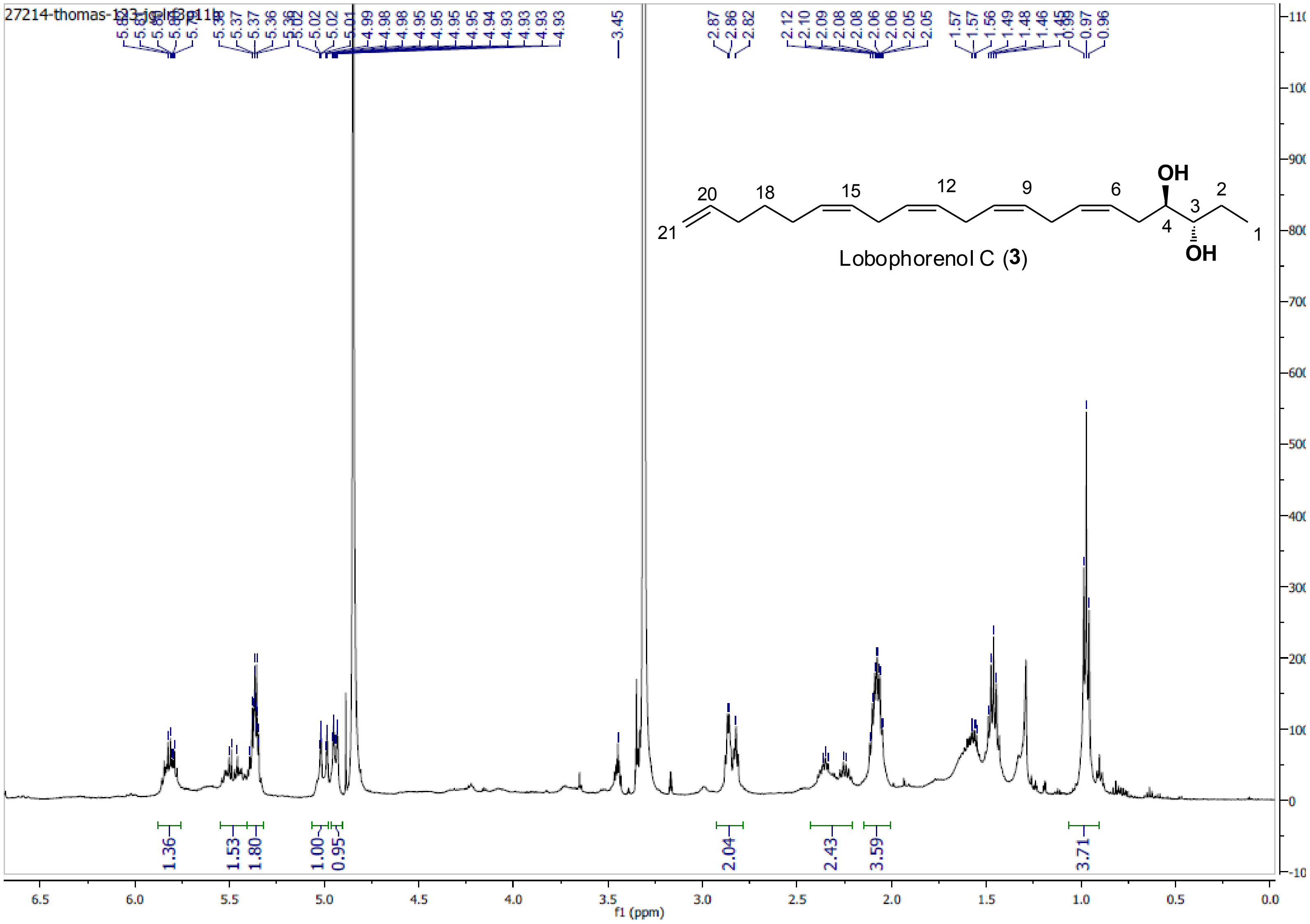

^{1}H NMR spectrum of **3** in CD_3OD (500 MHz)

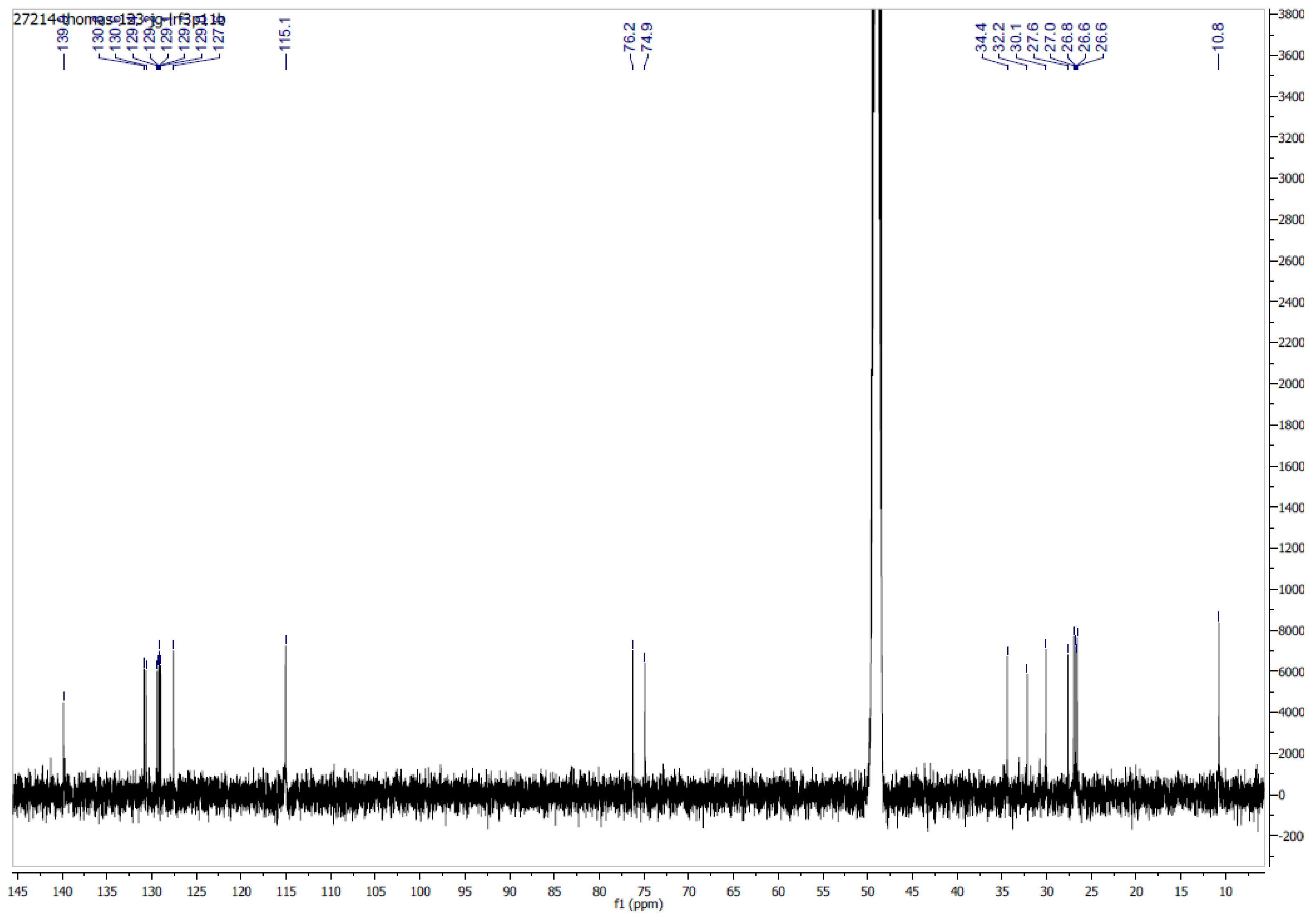

^{13}C NMR spectrum of **3** in CD_3OD (125 MHz)

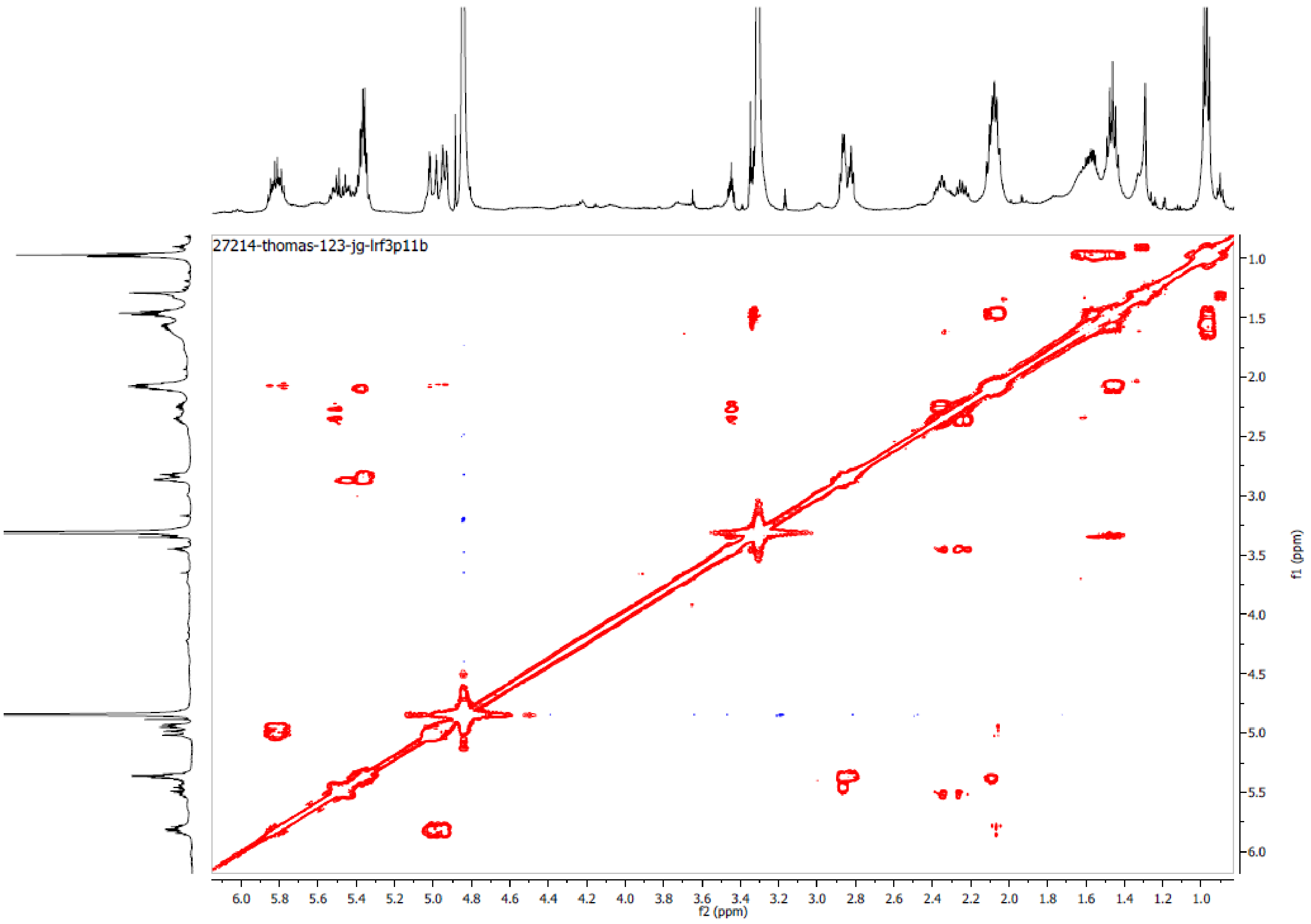

COSY NMR spectrum of **3** in CD_3OD (500 MHz)

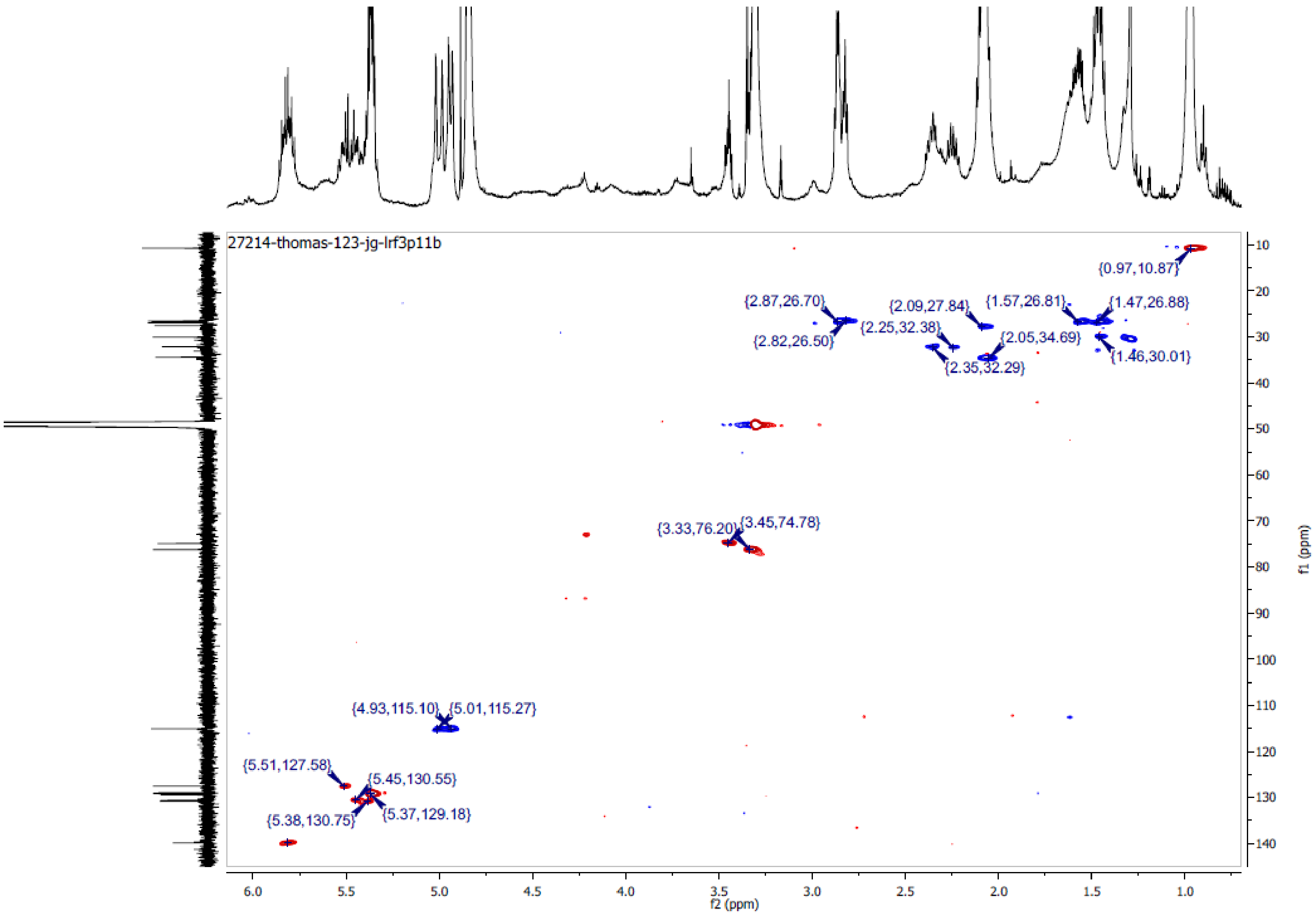

HSQC NMR spectrum of **3** in CD_3OD (500 MHz)

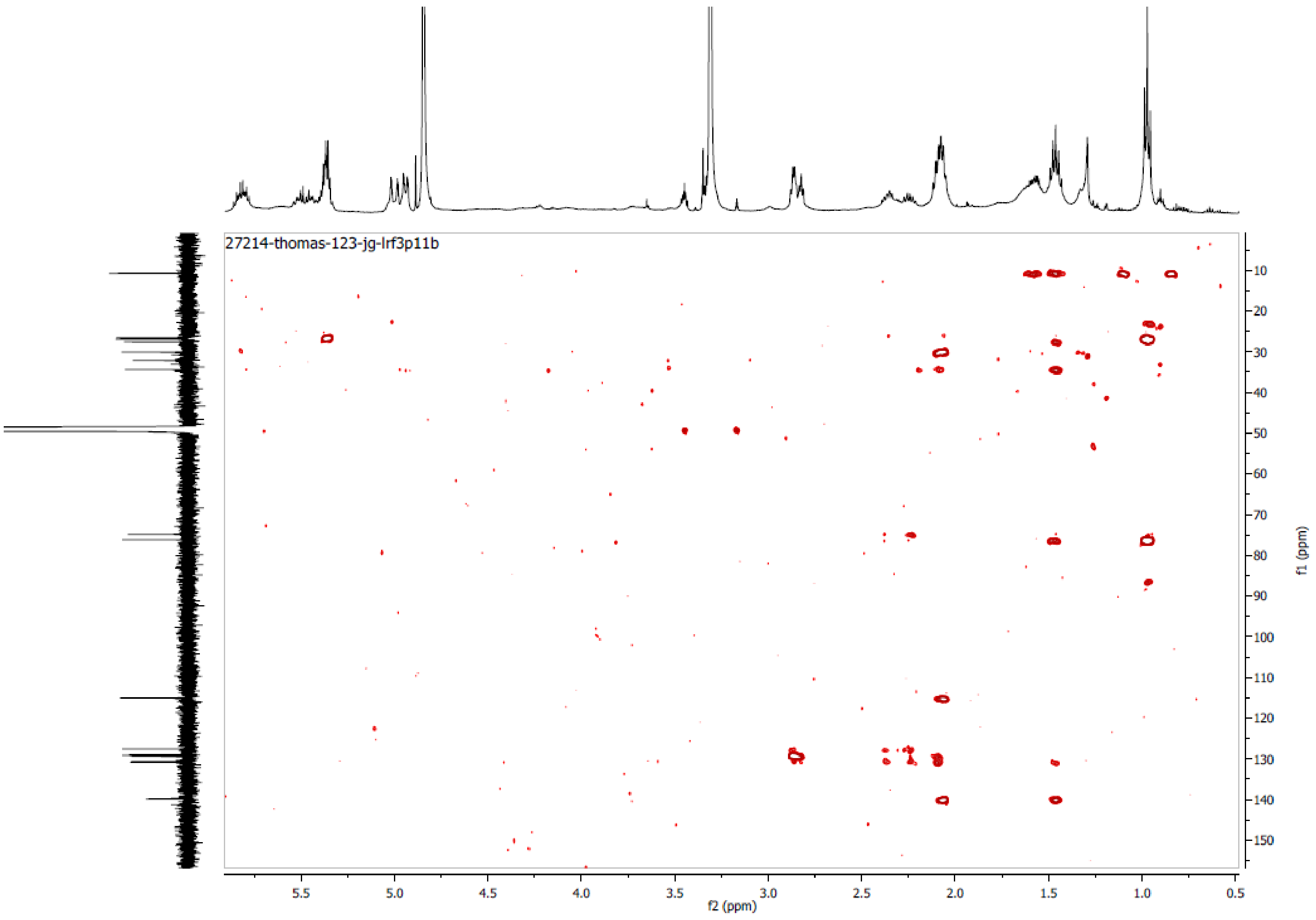

HMBC NMR spectrum of **3** in CD_3OD (500 MHz)

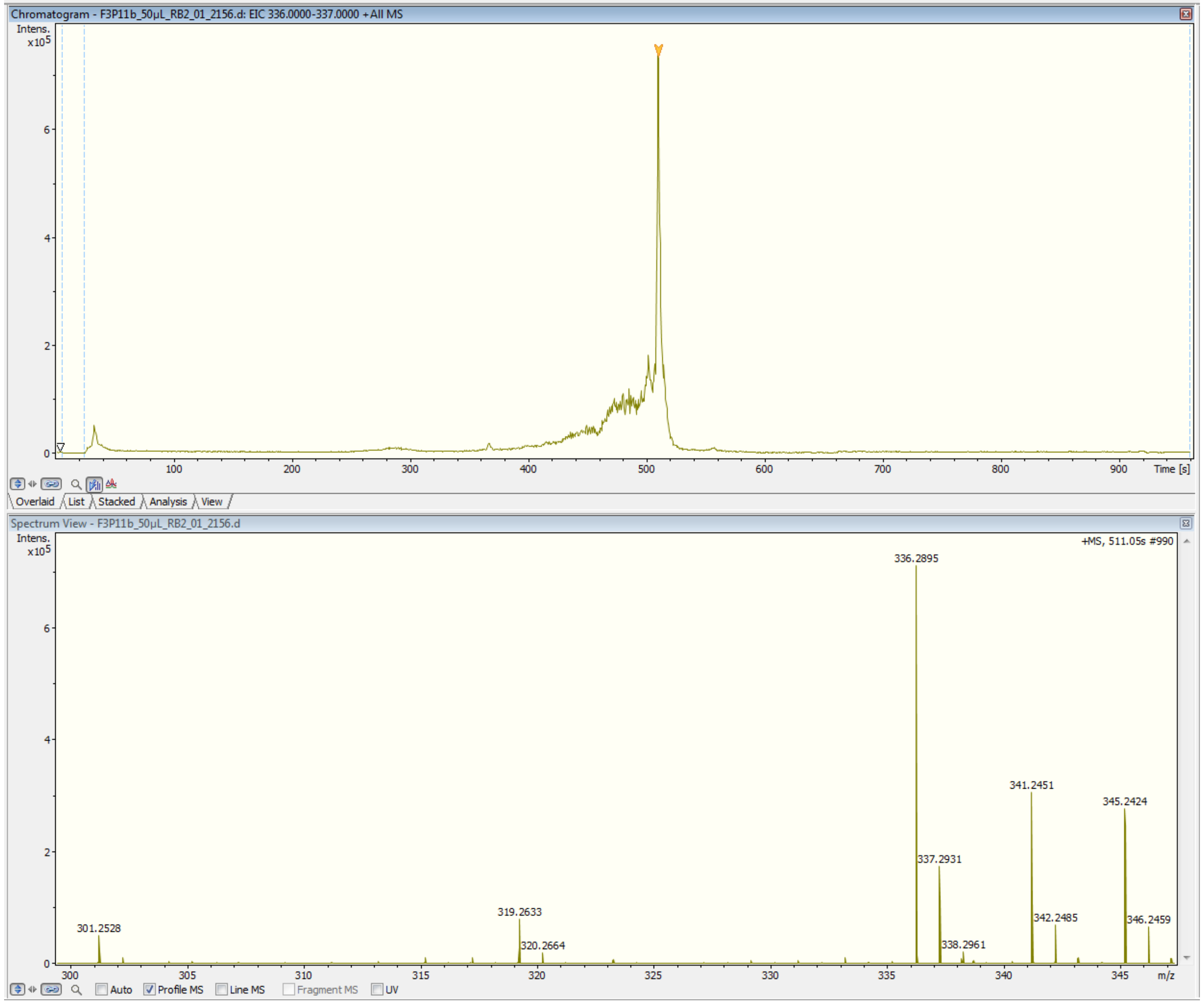
Chromatogram - F3P11b_50µL_RB2_01_2156.d: EIC 336.0000-337.0000 +All MS
Intens.
x10^5
Time [s]
Overlaid
List
Stacked
Analysis
View
Spectrum View - F3P11b_50µL_RB2_01_2156.d
+MS, 511.05s #990
336.2895
337.2931
338.2961
341.2451
342.2485
345.2424
346.2459
319.2633
320.2664
301.2528
m/z
Auto
Profile MS
Line MS
Fragment MS
UV

HR-(+)ESIMS of **3**

Assessment of the relative seafloor cover and quantification of *Lobophora*-corals association

Relative seafloor cover (i.e. corals, macroalgae, other benthic organisms) and quantification of *Lobophora*-corals association, assessed with the horizontal belt transect, was determined using the digital image processing software Coral Point Count with Excel extensions. Individual pictures were sub-divided into 4 rows and 4 columns, and each cell was populated with 1 random point lying within the cell borders. The circle with crosshairs with a diameter of 150 pixels was chosen as data point object shape. The feature lying under the circle-crosshairs was identified and recorded. If the crosshairs fell on a coral or *Lobophora*, the occurrence of the alga in direct contact with the coral within the circle was inspected and recorded.

Table S1. Sampling locations of *Lobophora* species in New Caledonia.

Species	Location	Reef type	Latitude	Longitude	Substrate
Lobophora abscondita	Bovis	Fringing reef	22° 13.488’S	166° 20.775’E	Coral
Lobophora crassa	Ricaudy	Fringing reef	22° 18.914’S	166° 27.453’E	Rubble
Lobophora dimorpha	Crouy	Patch reef	22° 21.919’S	166° 21.898’E	Coral
Lobophora hederacea	Abore	Inner barrier reef	22° 27.022’S	166° 22.441’E	Coral
Lobophora monticola	Saint Marie	Islet reef	22° 17.863’S	166° 28.898’E	Coral
Lobophora nigrescens	Crouy	Patch reef	22° 21.114’S	166° 21.084’E	Sand
Lobophora rosacea	Ricaudy	Fringing reef	22° 18.919’S	166° 27.463’E	Coral
Lobophora undulata	Larègnère	Islet reef	22° 19.548’S	166° 18.948’E	Coral

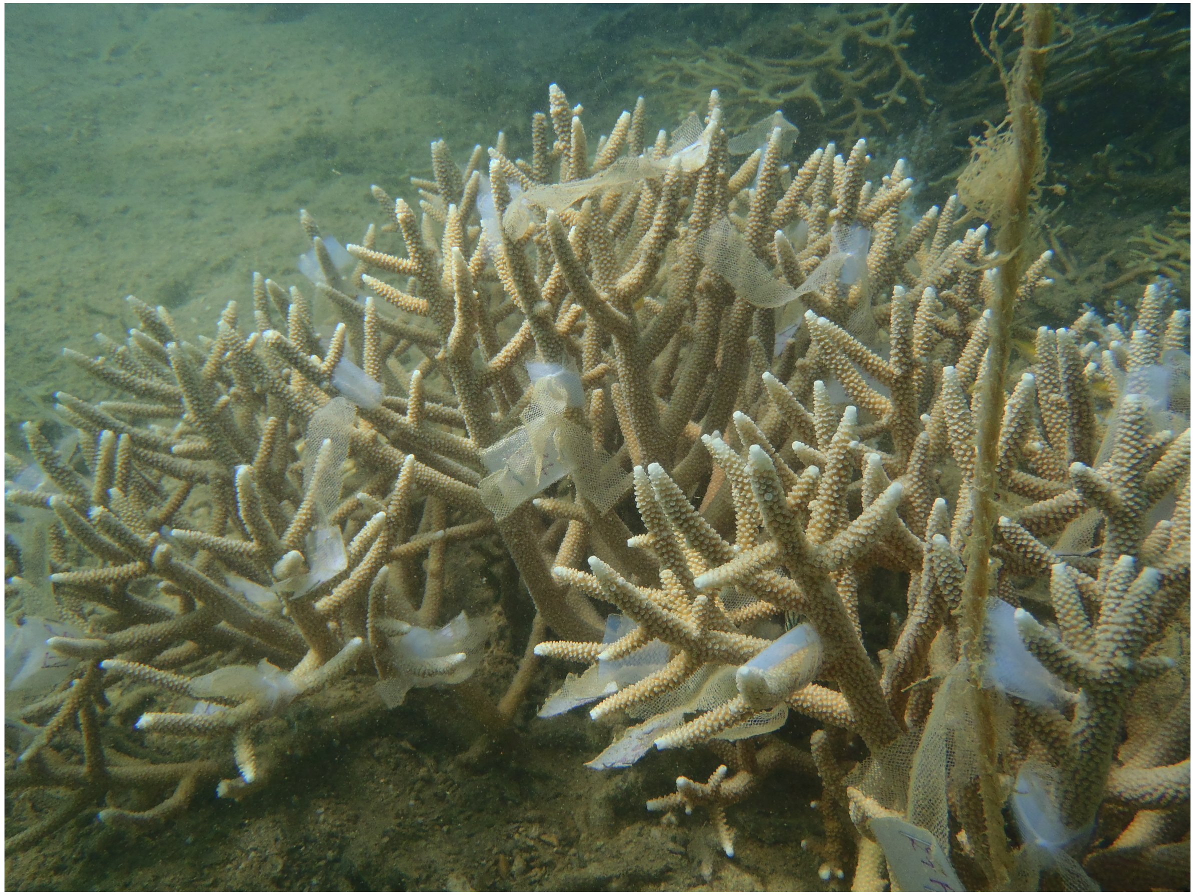

Figure S1. ***In situ*** **allelopathic bioassays. Photo credit: Christophe Vieira**
